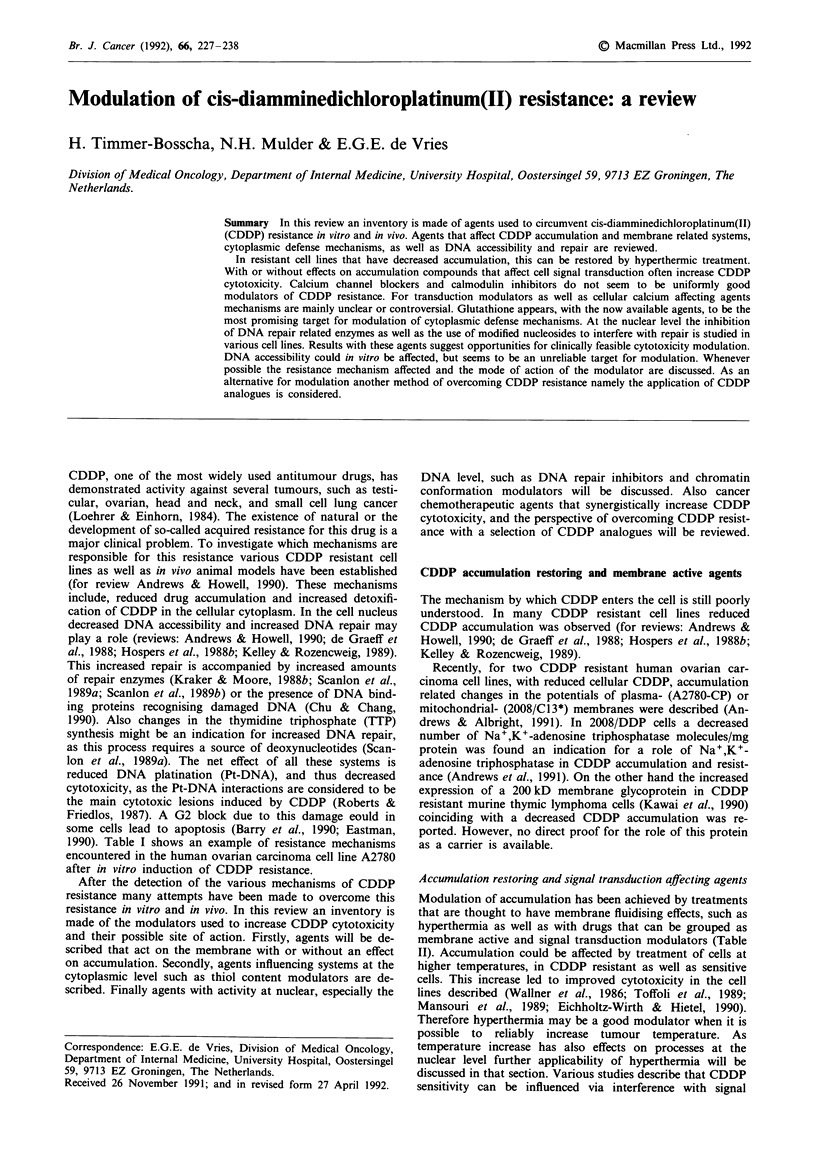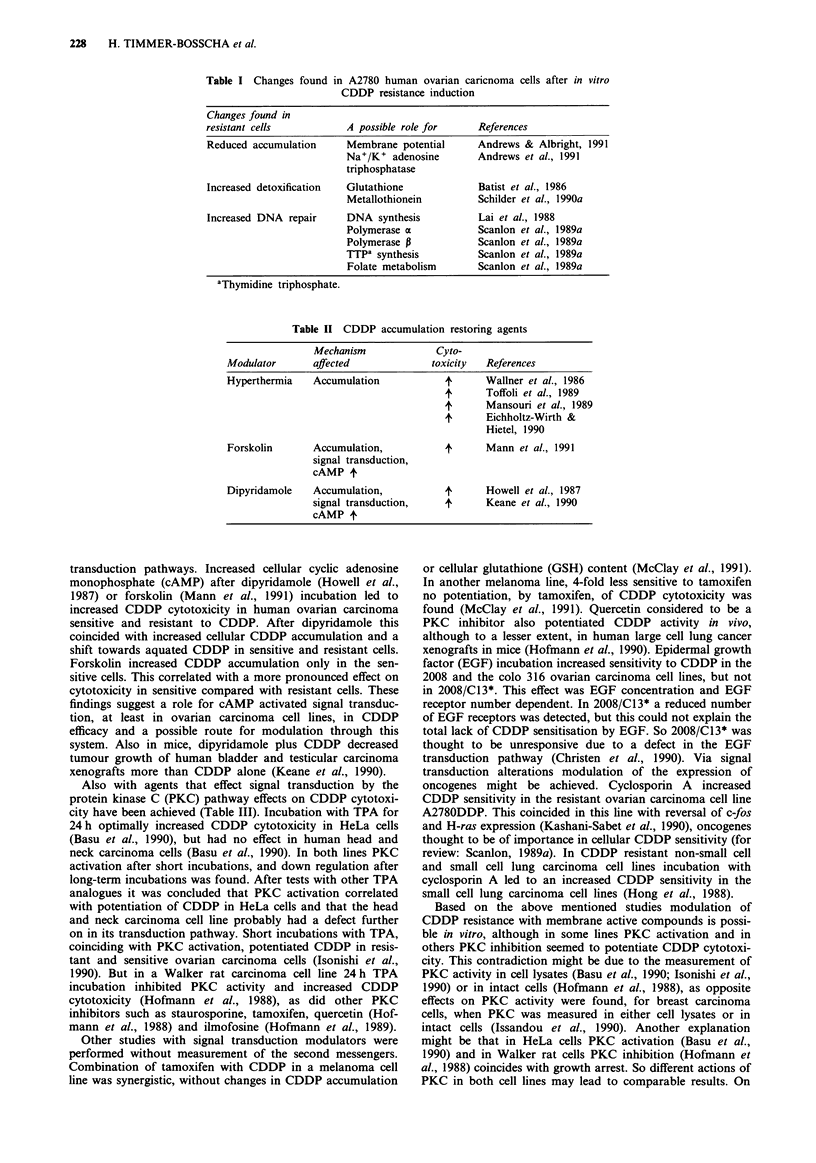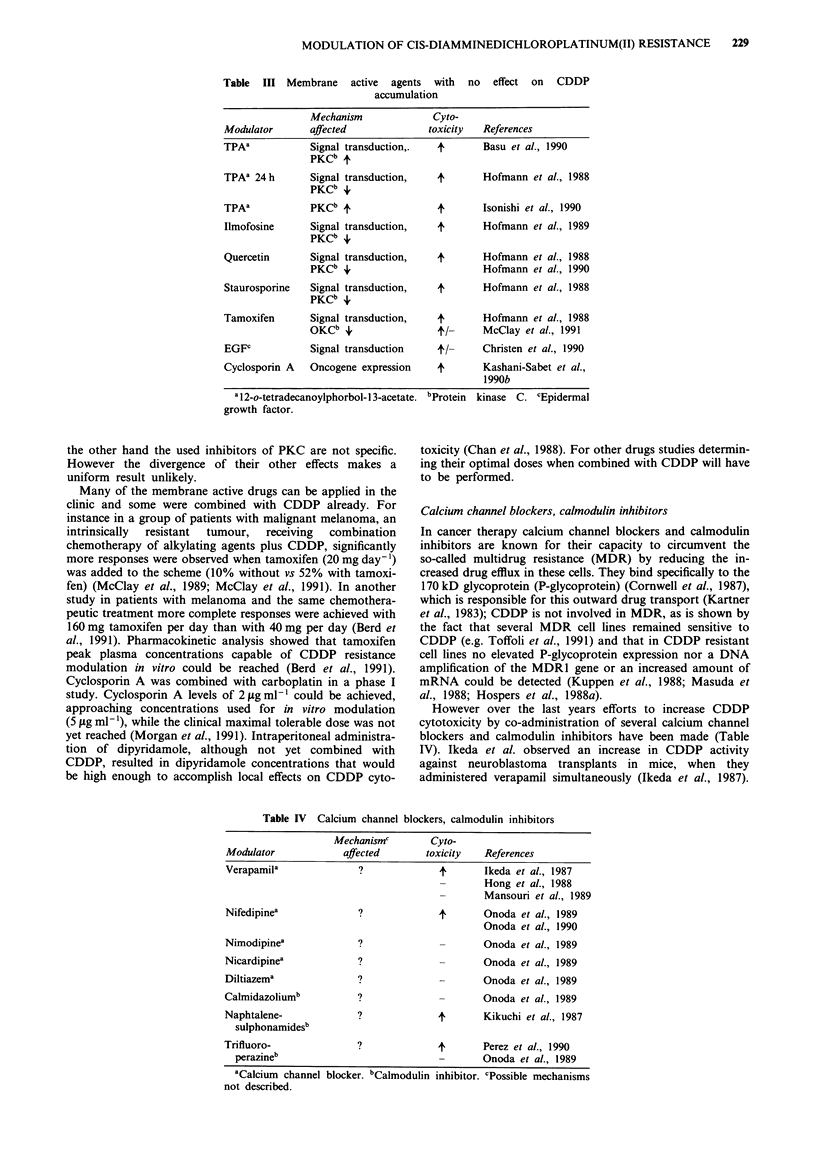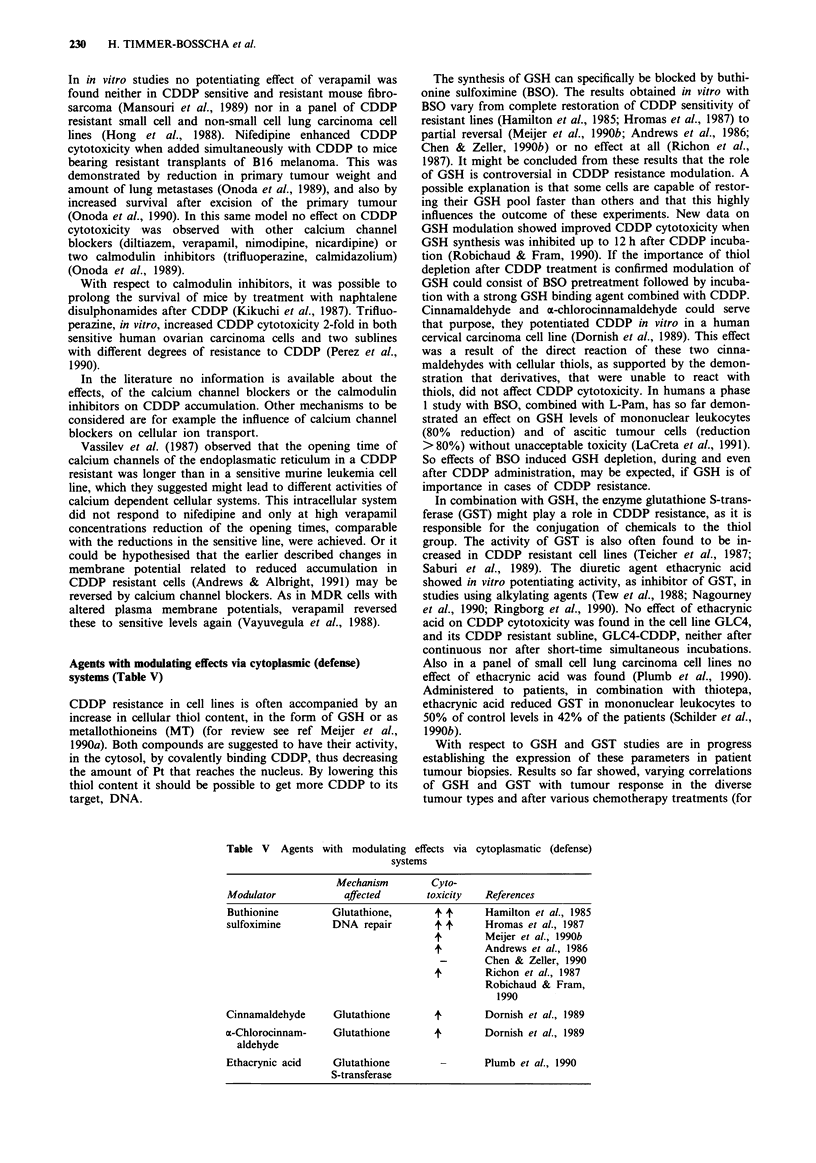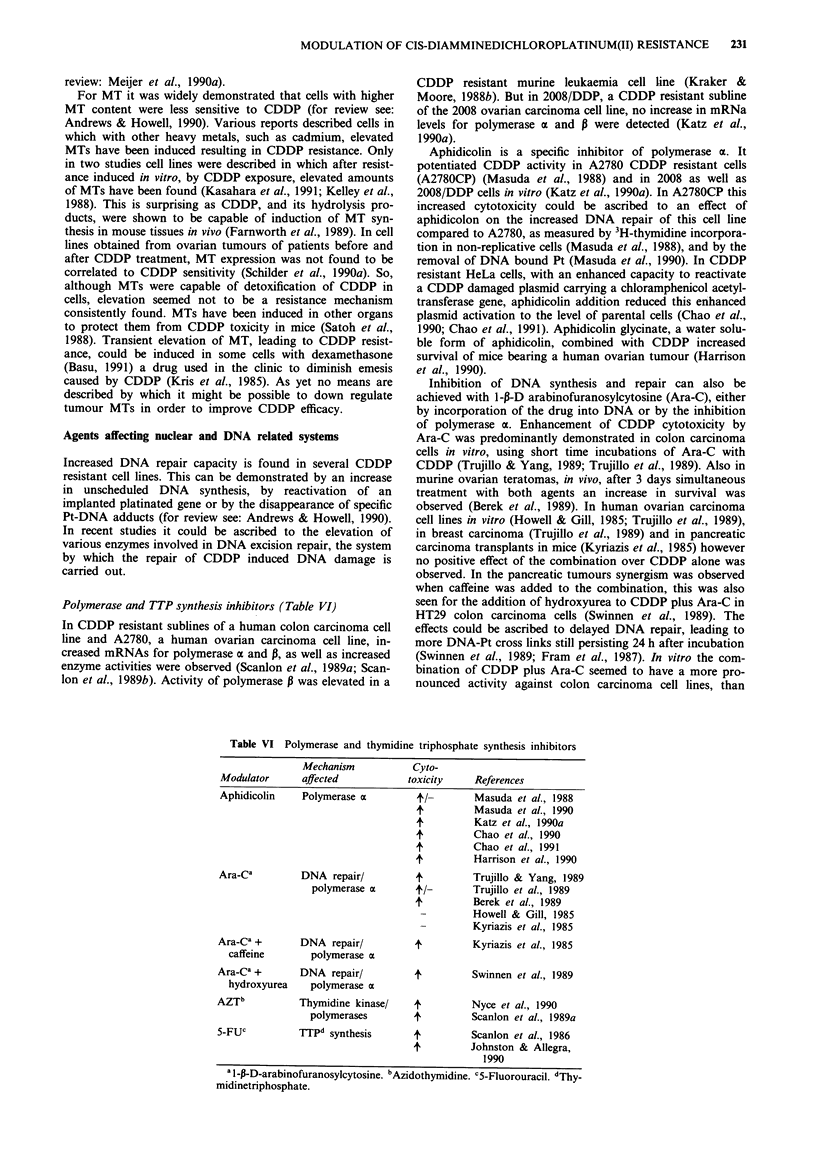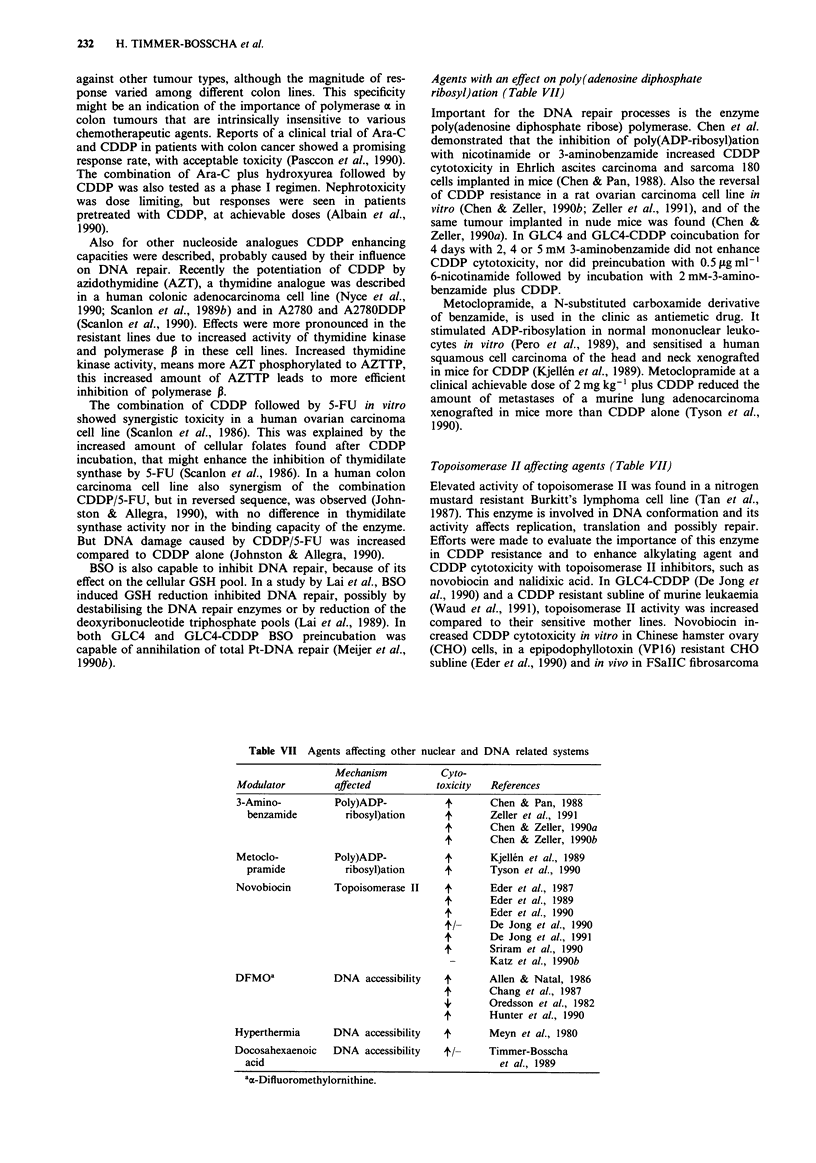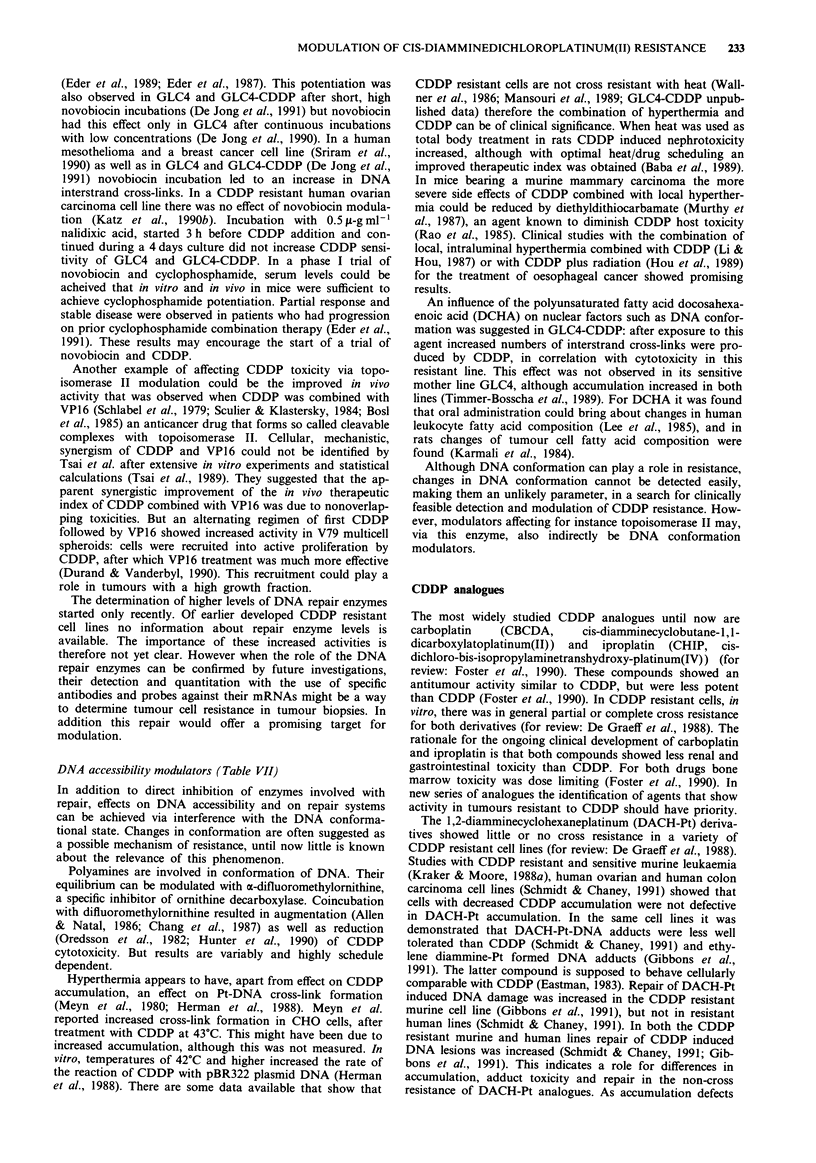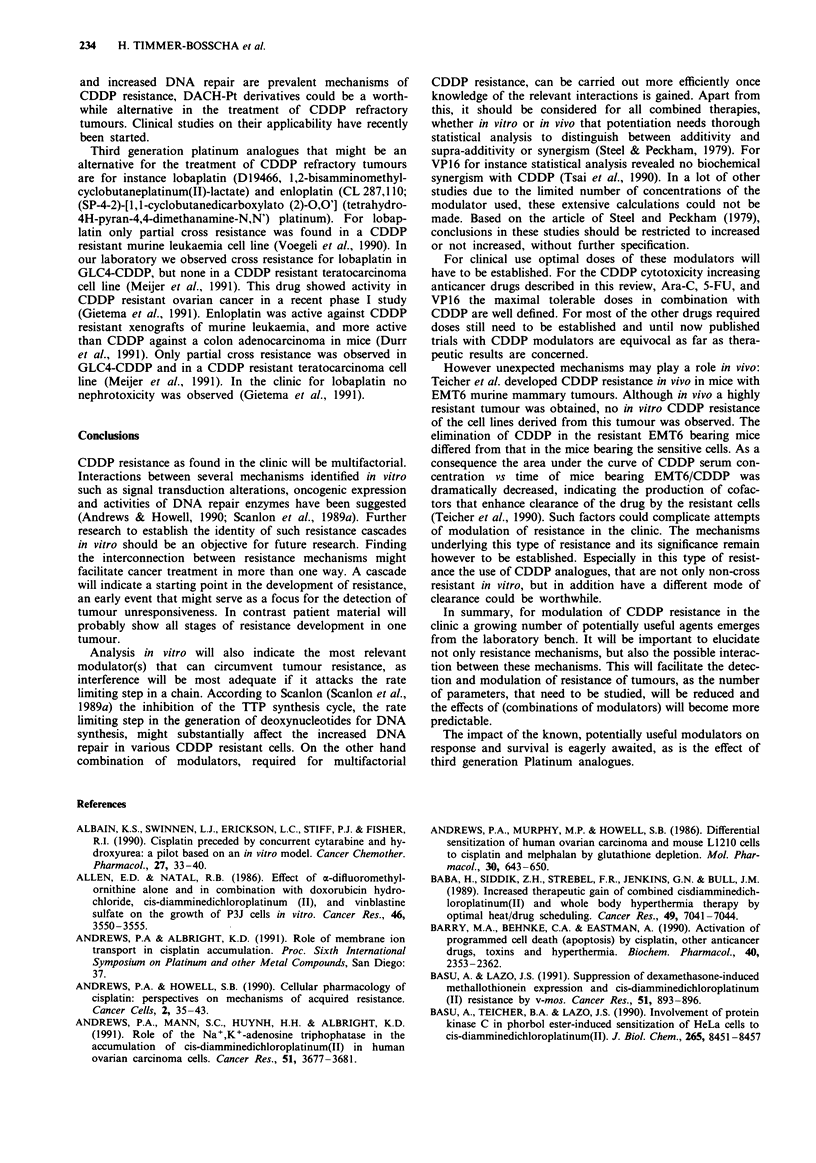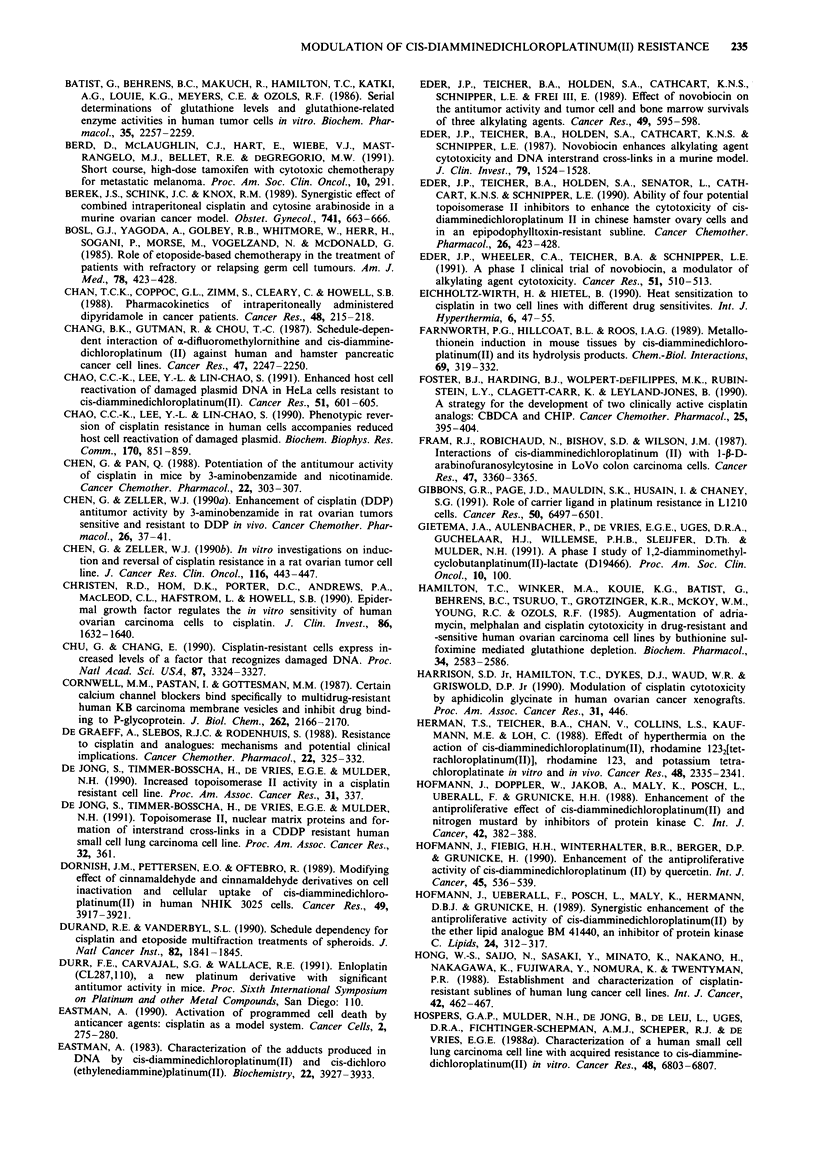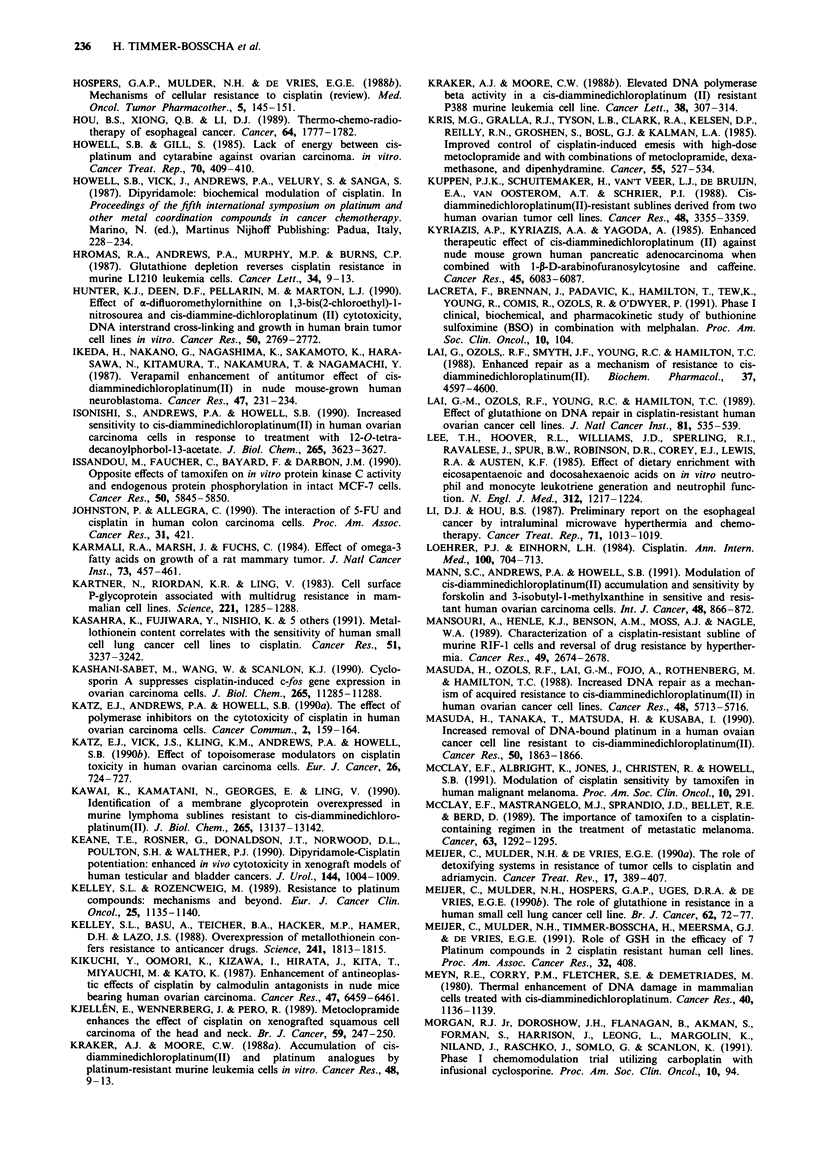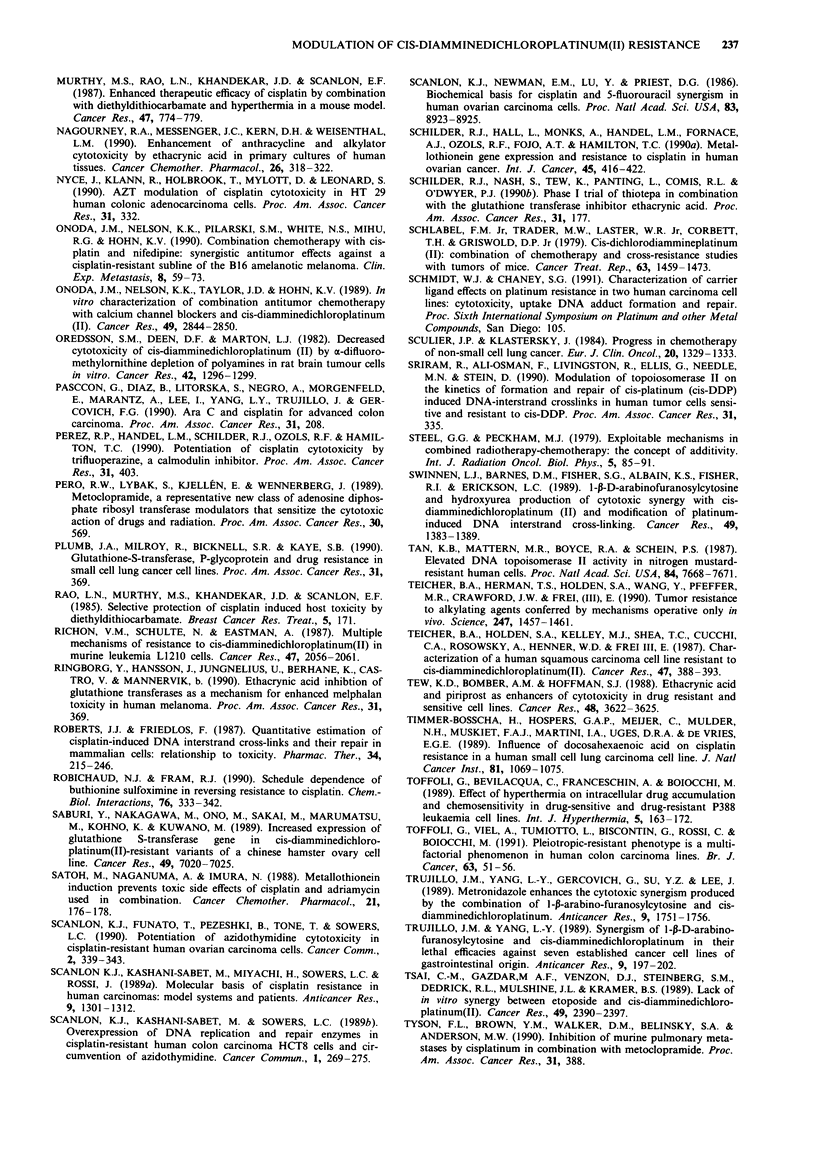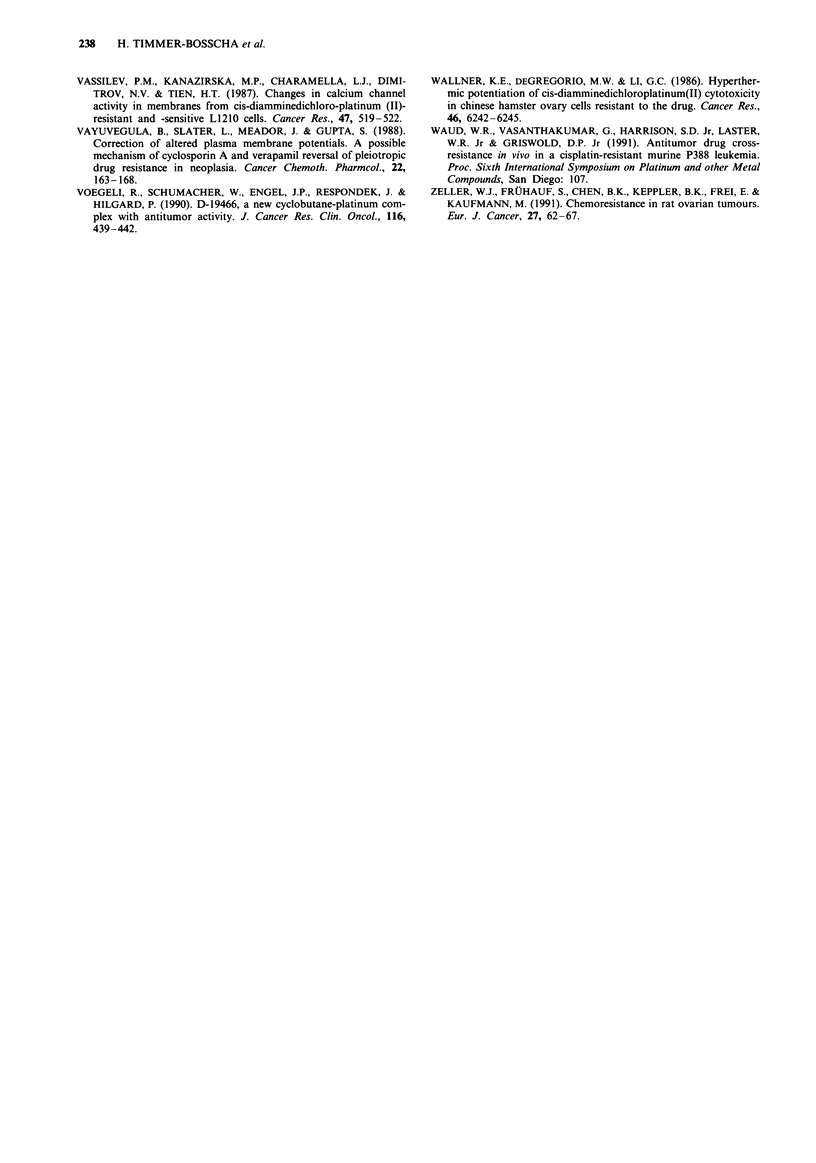# Modulation of cis-diamminedichloroplatinum(II) resistance: a review.

**DOI:** 10.1038/bjc.1992.249

**Published:** 1992-08

**Authors:** H. Timmer-Bosscha, N. H. Mulder, E. G. de Vries

**Affiliations:** Department of Internal Medicine, University Hospital, Groningen, The Netherlands.

## Abstract

In this review an inventory is made of agents used to circumvent cis-diamminedichloroplatinum(II) (CDDP) resistance in vitro and in vivo. Agents that affect CDDP accumulation and membrane related systems, cytoplasmic defense mechanisms, as well as DNA accessibility and repair are reviewed. In resistant cell lines that have decreased accumulation, this can be restored by hyperthermic treatment. With or without effects on accumulation compounds that affect cell signal transduction often increase CDDP cytotoxicity. Calcium channel blockers and calmodulin inhibitors do not seem to be uniformly good modulators of CDDP resistance. For transduction modulators as well as cellular calcium affecting agents mechanisms are mainly unclear or controversial. Glutathione appears, with the now available agents, to be the most promising target for modulation of cytoplasmic defense mechanisms. At the nuclear level the inhibition of DNA repair related enzymes as well as the use of modified nucleosides to interfere with repair is studied in various cell lines. Results with these agents suggest opportunities for clinically feasible cytotoxicity modulation. DNA accessibility could in vitro be affected, but seems to be an unreliable target for modulation. Whenever possible the resistance mechanism affected and the mode of action of the modulator are discussed. As an alternative for modulation another method of overcoming CDDP resistance namely the application of CDDP analogues is considered.


Br. J. Cancer (1992), 66, 227-238 © Macmillan Press Ltd., 1992

### Modulation of cis-diamminedichloroplatinum(II) resistance: a review

H. Timmer-Bosscha, N.H. Mulder & E.G.E. de Vries

*Division of Medical Oncology, Department of Internal Medicine, University Hospital, Oostersingel 59, 9713 EZ Groningen, The Netherlands.*

**Summary** In this review an inventory is made of agents used to circumvent cis-diamminedichloroplatinum(II) (CDDP) resistance *in vitro* and *in vivo*. Agents that affect CDDP accumulation and membrane related systems, cytoplasmic defense mechanisms, as well as DNA accessibility and repair are reviewed.

In resistant cell lines that have decreased accumulation, this can be restored by hyperthermic treatment. With or without effects on accumulation compounds that affect cell signal transduction often increase CDDP cytotoxicity. Calcium channel blockers and calmodulin inhibitors do not seem to be uniformly good modulators of CDDP resistance. For transduction modulators as well as cellular calcium affecting agents mechanisms are mainly unclear or controversial. Glutathione appears, with the now available agents, to be the most promising target for modulation of cytoplasmic defense mechanisms. At the nuclear level the inhibition of DNA repair related enzymes as well as the use of modified nucleosides to interfere with repair is studied in various cell lines. Results with these agents suggest opportunities for clinically feasible cytotoxicity modulation. DNA accessibility could *in vitro* be affected, but seems to be an unreliable target for modulation. Whenever possible the resistance mechanism affected and the mode of action of the modulator are discussed. As an alternative for modulation another method of overcoming CDDP resistance namely the application of CDDP analogues is considered.

CDDP, one of the most widely used antitumour drugs, has demonstrated activity against several tumours, such as testicular, ovarian, head and neck, and small cell lung cancer (Loehrer & Einhorn, 1984). The existence of natural or the development of so-called acquired resistance for this drug is a major clinical problem. To investigate which mechanisms are responsible for this resistance various CDDP resistant cell lines as well as *in vivo* animal models have been established (for review Andrews & Howell, 1990). These mechanisms include, reduced drug accumulation and increased detoxification of CDDP in the cellular cytoplasm. In the cell nucleus decreased DNA accessibility and increased DNA repair may play a role (reviews: Andrews & Howell, 1990; de Graeff *et al.*, 1988; Hospers *et al.*, 1988*b*; Kelley & Rozencweig, 1989). This increased repair is accompanied by increased amounts of repair enzymes (Kraker & Moore, 1988*b*; Scanlon *et al.*, 1989*a*; Scanlon *et al.*, 1989*b*) or the presence of DNA binding proteins recognising damaged DNA (Chu & Chang, 1990). Also changes in the thymidine triphosphate (TTP) synthesis might be an indication for increased DNA repair, as this process requires a source of deoxynucleotides (Scanlon *et al.*, 1989*a*). The net effect of all these systems is reduced DNA platination (Pt-DNA), and thus decreased cytotoxicity, as the Pt-DNA interactions are considered to be the main cytotoxic lesions induced by CDDP (Roberts & Friedlos, 1987). A G2 block due to this damage eould in some cells lead to apoptosis (Barry *et al.*, 1990; Eastman, 1990). Table I shows an example of resistance mechanisms encountered in the human ovarian carcinoma cell line A2780 after *in vitro* induction of CDDP resistance.

After the detection of the various mechanisms of CDDP resistance many attempts have been made to overcome this resistance *in vitro* and *in vivo*. In this review an inventory is made of the modulators used to increase CDDP cytotoxicity and their possible site of action. Firstly, agents will be described that act on the membrane with or without an effect on accumulation. Secondly, agents influencing systems at the cytoplasmic level such as thiol content modulators are described. Finally agents with activity at nuclear, especially the DNA level, such as DNA repair inhibitors and chromatin conformation modulators will be discussed. Also cancer chemotherapeutic agents that synergistically increase CDDP cytotoxicity, and the perspective of overcoming CDDP resistance with a selection of CDDP analogues will be reviewed.

Correspondence: E.G.E. de Vries, Division of Medical Oncology, Department of Internal Medicine, University Hospital, Oostersingel 59, 9713 EZ Groningen, The Netherlands.
Received 26 November 1991; and in revised form 27 April 1992.

### CDDP accumulation restoring and membrane active agents

The mechanism by which CDDP enters the cell is still poorly understood. In many CDDP resistant cell lines reduced CDDP accumulation was observed (for reviews: Andrews & Howell, 1990; de Graeff *et al.*, 1988; Hospers *et al.*, 1988*b*; Kelley & Rozencweig, 1989).

Recently, for two CDDP resistant human ovarian carcinoma cell lines, with reduced cellular CDDP, accumulation related changes in the potentials of plasma- (A2780-CP) or mitochondrial- (2008/C13*) membranes were described (Andrews & Albright, 1991). In 2008/DDP cells a decreased number of $Na^+$,$K^+$-adenosine triphosphatase molecules/mg protein was found an indication for a role of $Na^+$,$K^+$-adenosine triphosphatase in CDDP accumulation and resistance (Andrews *et al.*, 1991). On the other hand the increased expression of a 200 kD membrane glycoprotein in CDDP resistant murine thymic lymphoma cells (Kawai *et al.*, 1990) coinciding with a decreased CDDP accumulation was reported. However, no direct proof for the role of this protein as a carrier is available.

### *Accumulation restoring and signal transduction affecting agents*

Modulation of accumulation has been achieved by treatments that are thought to have membrane fluidising effects, such as hyperthermia as well as with drugs that can be grouped as membrane active and signal transduction modulators (Table II). Accumulation could be affected by treatment of cells at higher temperatures, in CDDP resistant as well as sensitive cells. This increase led to improved cytotoxicity in the cell lines described (Wallner *et al.*, 1986; Toffoli *et al.*, 1989; Mansouri *et al.*, 1989; Eichholtz-Wirth & Hietel, 1990). Therefore hyperthermia may be a good modulator when it is possible to reliably increase tumour temperature. As temperature increase has also effects on processes at the nuclear level further applicability of hyperthermia will be discussed in that section. Various studies describe that CDDP sensitivity can be influenced via interference with signal

**Table I** Changes found in A2780 human ovarian caricnoma cells after *in vitro* CDDP resistance induction

| *Changes found in resistant cells* | *A possible role for* | *References* |
|---|---|---|
| Reduced accumulation | Membrane potential | Andrews & Albright, 1991 |
| | $Na^+/K^+$ adenosine triphosphatase | Andrews *et al.*, 1991 |
| Increased detoxification | Glutathione | Batist *et al.*, 1986 |
| | Metallothionein | Schilder *et al.*, 1990*a* |
| Increased DNA repair | DNA synthesis | Lai *et al.*, 1988 |
| | Polymerase α | Scanlon *et al.*, 1989*a* |
| | Polymerase β | Scanlon *et al.*, 1989*a* |
| | TTP[a] synthesis | Scanlon *et al.*, 1989*a* |
| | Folate metabolism | Scanlon *et al.*, 1989*a* |

[a]Thymidine triphosphate.

**Table II** CDDP accumulation restoring agents

| *Modulator* | *Mechanism affected* | *Cyto-toxicity* | *References* |
|---|---|---|---|
| Hyperthermia | Accumulation | ↑ | Wallner *et al.*, 1986 |
| | | ↑ | Toffoli *et al.*, 1989 |
| | | ↑ | Mansouri *et al.*, 1989 |
| | | ↑ | Eichholtz-Wirth & Hietel, 1990 |
| Forskolin | Accumulation, signal transduction, cAMP ↑ | ↑ | Mann *et al.*, 1991 |
| Dipyridamole | Accumulation, signal transduction, cAMP ↑ | ↑ | Howell *et al.*, 1987 |
| | | ↑ | Keane *et al.*, 1990 |

transduction pathways. Increased cellular cyclic adenosine monophosphate (cAMP) after dipyridamole (Howell *et al.*, 1987) or forskolin (Mann *et al.*, 1991) incubation led to increased CDDP cytotoxicity in human ovarian carcinoma sensitive and resistant to CDDP. After dipyridamole this coincided with increased cellular CDDP accumulation and a shift towards aquated CDDP in sensitive and resistant cells. Forskolin increased CDDP accumulation only in the sensitive cells. This correlated with a more pronounced effect on cytotoxicity in sensitive compared with resistant cells. These findings suggest a role for cAMP activated signal transduction, at least in ovarian carcinoma cell lines, in CDDP efficacy and a possible route for modulation through this system. Also in mice, dipyridamole plus CDDP decreased tumour growth of human bladder and testicular carcinoma xenografts more than CDDP alone (Keane *et al.*, 1990).

Also with agents that effect signal transduction by the protein kinase C (PKC) pathway effects on CDDP cytotoxicity have been achieved (Table III). Incubation with TPA for 24 h optimally increased CDDP cytotoxicity in HeLa cells (Basu *et al.*, 1990), but had no effect in human head and neck carcinoma cells (Basu *et al.*, 1990). In both lines PKC activation after short incubations, and down regulation after long-term incubations was found. After tests with other TPA analogues it was concluded that PKC activation correlated with potentiation of CDDP in HeLa cells and that the head and neck carcinoma cell line probably had a defect further on in its transduction pathway. Short incubations with TPA, coinciding with PKC activation, potentiated CDDP in resistant and sensitive ovarian carcinoma cells (Isonishi *et al.*, 1990). But in a Walker rat carcinoma cell line 24 h TPA incubation inhibited PKC activity and increased CDDP cytotoxicity (Hofmann *et al.*, 1988), as did other PKC inhibitors such as staurosporine, tamoxifen, quercetin (Hofmann *et al.*, 1988) and ilmofosine (Hofmann *et al.*, 1989).

Other studies with signal transduction modulators were performed without measurement of the second messengers. Combination of tamoxifen with CDDP in a melanoma cell line was synergistic, without changes in CDDP accumulation or cellular glutathione (GSH) content (McClay *et al.*, 1991). In another melanoma line, 4-fold less sensitive to tamoxifen no potentiation, by tamoxifen, of CDDP cytotoxicity was found (McClay *et al.*, 1991). Quercetin considered to be a PKC inhibitor also potentiated CDDP activity *in vivo*, although to a lesser extent, in human large cell lung cancer xenografts in mice (Hofmann *et al.*, 1990). Epidermal growth factor (EGF) incubation increased sensitivity to CDDP in the 2008 and the colo 316 ovarian carcinoma cell lines, but not in 2008/C13*. This effect was EGF concentration and EGF receptor number dependent. In 2008/C13* a reduced number of EGF receptors was detected, but this could not explain the total lack of CDDP sensitisation by EGF. So 2008/C13* was thought to be unresponsive due to a defect in the EGF transduction pathway (Christen *et al.*, 1990). Via signal transduction alterations modulation of the expression of oncogenes might be achieved. Cyclosporin A increased CDDP sensitivity in the resistant ovarian carcinoma cell line A2780DDP. This coincided in this line with reversal of c-*fos* and H-*ras* expression (Kashani-Sabet *et al.*, 1990), oncogenes thought to be of importance in cellular CDDP sensitivity (for review: Scanlon, 1989*a*). In CDDP resistant non-small cell and small cell lung carcinoma cell lines incubation with cyclosporin A led to an increased CDDP sensitivity in the small cell lung carcinoma cell lines (Hong *et al.*, 1988).

Based on the above mentioned studies modulation of CDDP resistance with membrane active compounds is possible *in vitro*, although in some lines PKC activation and in others PKC inhibition seemed to potentiate CDDP cytotoxicity. This contradiction might be due to the measurement of PKC activity in cell lysates (Basu *et al.*, 1990; Isonishi *et al.*, 1990) or in intact cells (Hofmann *et al.*, 1988), as opposite effects on PKC activity were found, for breast carcinoma cells, when PKC was measured in either cell lysates or in intact cells (Issandou *et al.*, 1990). Another explanation might be that in HeLa cells PKC activation (Basu *et al.*, 1990) and in Walker rat cells PKC inhibition (Hofmann *et al.*, 1988) coincides with growth arrest. So different actions of PKC in both cell lines may lead to comparable results. On

**Table III** Membrane active agents with no effect on CDDP accumulation

| *Modulator* | *Mechanism affected* | *Cyto-toxicity* | *References* |
|---|---|---|---|
| TPA[a] | Signal transduction,. PKC[b] ↑ | ↑ | Basu *et al.*, 1990 |
| TPA[a] 24 h | Signal transduction, PKC[b] ↓ | ↑ | Hofmann *et al.*, 1988 |
| TPA[a] | PKC[b] ↑ | ↑ | Isonishi *et al.*, 1990 |
| Ilmofosine | Signal transduction, PKC[b] ↓ | ↑ | Hofmann *et al.*, 1989 |
| Quercetin | Signal transduction, PKC[b] ↓ | ↑ | Hofmann *et al.*, 1988<br>Hofmann *et al.*, 1990 |
| Staurosporine | Signal transduction, PKC[b] ↓ | ↑ | Hofmann *et al.*, 1988 |
| Tamoxifen | Signal transduction, OKC[b] ↓ | ↑<br>↑/– | Hofmann *et al.*, 1988<br>McClay *et al.*, 1991 |
| EGF[c] | Signal transduction | ↑/– | Christen *et al.*, 1990 |
| Cyclosporin A | Oncogene expression | ↑ | Kashani-Sabet *et al.*, 1990*b* |

[a]12-*o*-tetradecanoylphorbol-13-acetate. [b]Protein kinase C. [c]Epidermal growth factor.

the other hand the used inhibitors of PKC are not specific. However the divergence of their other effects makes a uniform result unlikely.

Many of the membrane active drugs can be applied in the clinic and some were combined with CDDP already. For instance in a group of patients with malignant melanoma, an intrinsically resistant tumour, receiving combination chemotherapy of alkylating agents plus CDDP, significantly more responses were observed when tamoxifen (20 mg $day^{-1}$) was added to the scheme (10% without *vs* 52% with tamoxifen) (McClay *et al.*, 1989; McClay *et al.*, 1991). In another study in patients with melanoma and the same chemotherapeutic treatment more complete responses were achieved with 160 mg tamoxifen per day than with 40 mg per day (Berd *et al.*, 1991). Pharmacokinetic analysis showed that tamoxifen peak plasma concentrations capable of CDDP resistance modulation *in vitro* could be reached (Berd *et al.*, 1991). Cyclosporin A was combined with carboplatin in a phase I study. Cyclosporin A levels of 2 $\mu g\ ml^{-1}$ could be achieved, approaching concentrations used for *in vitro* modulation (5 $\mu g\ ml^{-1}$), while the clinical maximal tolerable dose was not yet reached (Morgan *et al.*, 1991). Intraperitoneal administration of dipyridamole, although not yet combined with CDDP, resulted in dipyridamole concentrations that would be high enough to accomplish local effects on CDDP cytotoxicity (Chan *et al.*, 1988). For other drugs studies determining their optimal doses when combined with CDDP will have to be performed.

*Calcium channel blockers, calmodulin inhibitors*

In cancer therapy calcium channel blockers and calmodulin inhibitors are known for their capacity to circumvent the so-called multidrug resistance (MDR) by reducing the increased drug efflux in these cells. They bind specifically to the 170 kD glycoprotein (P-glycoprotein) (Cornwell *et al.*, 1987), which is responsible for this outward drug transport (Kartner *et al.*, 1983); CDDP is not involved in MDR, as is shown by the fact that several MDR cell lines remained sensitive to CDDP (e.g. Toffoli *et al.*, 1991) and that in CDDP resistant cell lines no elevated P-glycoprotein expression nor a DNA amplification of the MDR1 gene or an increased amount of mRNA could be detected (Kuppen *et al.*, 1988; Masuda *et al.*, 1988; Hospers *et al.*, 1988*a*).

However over the last years efforts to increase CDDP cytotoxicity by co-administration of several calcium channel blockers and calmodulin inhibitors have been made (Table IV). Ikeda *et al.* observed an increase in CDDP activity against neuroblastoma transplants in mice, when they administered verapamil simultaneously (Ikeda *et al.*, 1987).

**Table IV** Calcium channel blockers, calmodulin inhibitors

| *Modulator* | *Mechanism[c] affected* | *Cyto-toxicity* | *References* |
|---|---|---|---|
| Verapamil[a] | ? | ↑<br>–<br>– | Ikeda *et al.*, 1987<br>Hong *et al.*, 1988<br>Mansouri *et al.*, 1989 |
| Nifedipine[a] | ? | ↑ | Onoda *et al.*, 1989<br>Onoda *et al.*, 1990 |
| Nimodipine[a] | ? | – | Onoda *et al.*, 1989 |
| Nicardipine[a] | ? | – | Onoda *et al.*, 1989 |
| Diltiazem[a] | ? | – | Onoda *et al.*, 1989 |
| Calmidazolium[b] | ? | – | Onoda *et al.*, 1989 |
| Naphtalene-sulphonamides[b] | ? | ↑ | Kikuchi *et al.*, 1987 |
| Trifluoro-perazine[b] | ? | ↑<br>– | Perez *et al.*, 1990<br>Onoda *et al.*, 1989 |

[a]Calcium channel blocker. [b]Calmodulin inhibitor. [c]Possible mechanisms not described.

In *in vitro* studies no potentiating effect of verapamil was found neither in CDDP sensitive and resistant mouse fibrosarcoma (Mansouri *et al.*, 1989) nor in a panel of CDDP resistant small cell and non-small cell lung carcinoma cell lines (Hong *et al.*, 1988). Nifedipine enhanced CDDP cytotoxicity when added simultaneously with CDDP to mice bearing resistant transplants of B16 melanoma. This was demonstrated by reduction in primary tumour weight and amount of lung metastases (Onoda *et al.*, 1989), and also by increased survival after excision of the primary tumour (Onoda *et al.*, 1990). In this same model no effect on CDDP cytotoxicity was observed with other calcium channel blockers (diltiazem, verapamil, nimodipine, nicardipine) or two calmodulin inhibitors (trifluoperazine, calmidazolium) (Onoda *et al.*, 1989).

With respect to calmodulin inhibitors, it was possible to prolong the survival of mice by treatment with naphtalene disulphonamides after CDDP (Kikuchi *et al.*, 1987). Trifluoperazine, *in vitro*, increased CDDP cytotoxicity 2-fold in both sensitive human ovarian carcinoma cells and two sublines with different degrees of resistance to CDDP (Perez *et al.*, 1990).

In the literature no information is available about the effects, of the calcium channel blockers or the calmodulin inhibitors on CDDP accumulation. Other mechanisms to be considered are for example the influence of calcium channel blockers on cellular ion transport.

Vassilev *et al.* (1987) observed that the opening time of calcium channels of the endoplasmatic reticulum in a CDDP resistant was longer than in a sensitive murine leukemia cell line, which they suggested might lead to different activities of calcium dependent cellular systems. This intracellular system did not respond to nifedipine and only at high verapamil concentrations reduction of the opening times, comparable with the reductions in the sensitive line, were achieved. Or it could be hypothesised that the earlier described changes in membrane potential related to reduced accumulation in CDDP resistant cells (Andrews & Albright, 1991) may be reversed by calcium channel blockers. As in MDR cells with altered plasma membrane potentials, verapamil reversed these to sensitive levels again (Vayuvegula *et al.*, 1988).

### Agents with modulating effects via cytoplasmic (defense) systems (Table V)

CDDP resistance in cell lines is often accompanied by an increase in cellular thiol content, in the form of GSH or as metallothioneins (MT) (for review see ref Meijer *et al.*, 1990*a*). Both compounds are suggested to have their activity, in the cytosol, by covalently binding CDDP, thus decreasing the amount of Pt that reaches the nucleus. By lowering this thiol content it should be possible to get more CDDP to its target, DNA.

The synthesis of GSH can specifically be blocked by buthionine sulfoximine (BSO). The results obtained *in vitro* with BSO vary from complete restoration of CDDP sensitivity of resistant lines (Hamilton *et al.*, 1985; Hromas *et al.*, 1987) to partial reversal (Meijer *et al.*, 1990*b*; Andrews *et al.*, 1986; Chen & Zeller, 1990*b*) or no effect at all (Richon *et al.*, 1987). It might be concluded from these results that the role of GSH is controversial in CDDP resistance modulation. A possible explanation is that some cells are capable of restoring their GSH pool faster than others and that this highly influences the outcome of these experiments. New data on GSH modulation showed improved CDDP cytotoxicity when GSH synthesis was inhibited up to 12 h after CDDP incubation (Robichaud & Fram, 1990). If the importance of thiol depletion after CDDP treatment is confirmed modulation of GSH could consist of BSO pretreatment followed by incubation with a strong GSH binding agent combined with CDDP. Cinnamaldehyde and α-chlorocinnamaldehyde could serve that purpose, they potentiated CDDP *in vitro* in a human cervical carcinoma cell line (Dornish *et al.*, 1989). This effect was a result of the direct reaction of these two cinnamaldehydes with cellular thiols, as supported by the demonstration that derivatives, that were unable to react with thiols, did not affect CDDP cytotoxicity. In humans a phase 1 study with BSO, combined with L-Pam, has so far demonstrated an effect on GSH levels of mononuclear leukocytes (80% reduction) and of ascitic tumour cells (reduction >80%) without unacceptable toxicity (LaCreta *et al.*, 1991). So effects of BSO induced GSH depletion, during and even after CDDP administration, may be expected, if GSH is of importance in cases of CDDP resistance.

In combination with GSH, the enzyme glutathione S-transferase (GST) might play a role in CDDP resistance, as it is responsible for the conjugation of chemicals to the thiol group. The activity of GST is also often found to be increased in CDDP resistant cell lines (Teicher *et al.*, 1987; Saburi *et al.*, 1989). The diuretic agent ethacrynic acid showed *in vitro* potentiating activity, as inhibitor of GST, in studies using alkylating agents (Tew *et al.*, 1988; Nagourney *et al.*, 1990; Ringborg *et al.*, 1990). No effect of ethacrynic acid on CDDP cytotoxicity was found in the cell line GLC4, and its CDDP resistant subline, GLC4-CDDP, neither after continuous nor after short-time simultaneous incubations. Also in a panel of small cell lung carcinoma cell lines no effect of ethacrynic acid was found (Plumb *et al.*, 1990). Administered to patients, in combination with thiotepa, ethacrynic acid reduced GST in mononuclear leukocytes to 50% of control levels in 42% of the patients (Schilder *et al.*, 1990*b*).

With respect to GSH and GST studies are in progress establishing the expression of these parameters in patient tumour biopsies. Results so far showed, varying correlations of GSH and GST with tumour response in the diverse tumour types and after various chemotherapy treatments (for

**Table V** Agents with modulating effects via cytoplasmatic (defense) systems

| *Modulator* | *Mechanism affected* | *Cytotoxicity* | *References* |
|---|---|---|---|
| Buthionine sulfoximine | Glutathione, DNA repair | ↑↑ | Hamilton *et al.*, 1985 |
| | | ↑↑ | Hromas *et al.*, 1987 |
| | | ↑ | Meijer *et al.*, 1990*b* |
| | | ↑ | Andrews *et al.*, 1986 |
| | | – | Chen & Zeller, 1990 |
| | | ↑ | Richon *et al.*, 1987 |
| | | | Robichaud & Fram, 1990 |
| Cinnamaldehyde | Glutathione | ↑ | Dornish *et al.*, 1989 |
| α-Chlorocinnamaldehyde | Glutathione | ↑ | Dornish *et al.*, 1989 |
| Ethacrynic acid | Glutathione S-transferase | – | Plumb *et al.*, 1990 |

review: Meijer *et al.*, 1990*a*).

For MT it was widely demonstrated that cells with higher MT content were less sensitive to CDDP (for review see: Andrews & Howell, 1990). Various reports described cells in which with other heavy metals, such as cadmium, elevated MTs have been induced resulting in CDDP resistance. Only in two studies cell lines were described in which after resistance induced *in vitro*, by CDDP exposure, elevated amounts of MTs have been found (Kasahara *et al.*, 1991; Kelley *et al.*, 1988). This is surprising as CDDP, and its hydrolysis products, were shown to be capable of induction of MT synthesis in mouse tissues *in vivo* (Farnworth *et al.*, 1989). In cell lines obtained from ovarian tumours of patients before and after CDDP treatment, MT expression was not found to be correlated to CDDP sensitivity (Schilder *et al.*, 1990*a*). So, although MTs were capable of detoxification of CDDP in cells, elevation seemed not to be a resistance mechanism consistently found. MTs have been induced in other organs to protect them from CDDP toxicity in mice (Satoh *et al.*, 1988). Transient elevation of MT, leading to CDDP resistance, could be induced in some cells with dexamethasone (Basu, 1991) a drug used in the clinic to diminish emesis caused by CDDP (Kris *et al.*, 1985). As yet no means are described by which it might be possible to down regulate tumour MTs in order to improve CDDP efficacy.

### Agents affecting nuclear and DNA related systems

Increased DNA repair capacity is found in several CDDP resistant cell lines. This can be demonstrated by an increase in unscheduled DNA synthesis, by reactivation of an implanted platinated gene or by the disappearance of specific Pt-DNA adducts (for review see: Andrews & Howell, 1990). In recent studies it could be ascribed to the elevation of various enzymes involved in DNA excision repair, the system by which the repair of CDDP induced DNA damage is carried out.

### *Polymerase and TTP synthesis inhibitors (Table VI)*

In CDDP resistant sublines of a human colon carcinoma cell line and A2780, a human ovarian carcinoma cell line, increased mRNAs for polymerase α and β, as well as increased enzyme activities were observed (Scanlon *et al.*, 1989*a*; Scanlon *et al.*, 1989*b*). Activity of polymerase β was elevated in a CDDP resistant murine leukaemia cell line (Kraker & Moore, 1988*b*). But in 2008/DDP, a CDDP resistant subline of the 2008 ovarian carcinoma cell line, no increase in mRNa levels for polymerase α and β were detected (Katz *et al.*, 1990*a*).

Aphidicolin is a specific inhibitor of polymerase α. It potentiated CDDP activity in A2780 CDDP resistant cells (A2780CP) (Masuda *et al.*, 1988) and in 2008 as well as 2008/DDP cells *in vitro* (Katz *et al.*, 1990*a*). In A2780CP this increased cytotoxicity could be ascribed to an effect of aphidicolon on the increased DNA repair of this cell line compared to A2780, as measured by $^{3}$H-thymidine incorporation in non-replicative cells (Masuda *et al.*, 1988), and by the removal of DNA bound Pt (Masuda *et al.*, 1990). In CDDP resistant HeLa cells, with an enhanced capacity to reactivate a CDDP damaged plasmid carrying a chloramphenicol acetyltransferase gene, aphidicolin addition reduced this enhanced plasmid activation to the level of parental cells (Chao *et al.*, 1990; Chao *et al.*, 1991). Aphidicolin glycinate, a water soluble form of aphidicolin, combined with CDDP increased survival of mice bearing a human ovarian tumour (Harrison *et al.*, 1990).

Inhibition of DNA synthesis and repair can also be achieved with 1-β-D arabinofuranosylcytosine (Ara-C), either by incorporation of the drug into DNA or by the inhibition of polymerase α. Enhancement of CDDP cytotoxicity by Ara-C was predominantly demonstrated in colon carcinoma cells *in vitro*, using short time incubations of Ara-C with CDDP (Trujillo & Yang, 1989; Trujillo *et al.*, 1989). Also in murine ovarian teratomas, *in vivo*, after 3 days simultaneous treatment with both agents an increase in survival was observed (Berek *et al.*, 1989). In human ovarian carcinoma cell lines *in vitro* (Howell & Gill, 1985; Trujillo *et al.*, 1989), in breast carcinoma (Trujillo *et al.*, 1989) and in pancreatic carcinoma transplants in mice (Kyriazis *et al.*, 1985) however no positive effect of the combination over CDDP alone was observed. In the pancreatic tumours synergism was observed when caffeine was added to the combination, this was also seen for the addition of hydroxyurea to CDDP plus Ara-C in HT29 colon carcinoma cells (Swinnen *et al.*, 1989). The effects could be ascribed to delayed DNA repair, leading to more DNA-Pt cross links still persisting 24 h after incubation (Swinnen *et al.*, 1989; Fram *et al.*, 1987). *In vitro* the combination of CDDP plus Ara-C seemed to have a more pronounced activity against colon carcinoma cell lines, than

**Table VI** Polymerase and thymidine triphosphate synthesis inhibitors

| *Modulator* | *Mechanism affected* | *Cytotoxicity* | *References* |
|---|---|---|---|
| Aphidicolin | Polymerase α | ↑/– | Masuda *et al.*, 1988 |
| | | ↑ | Masuda *et al.*, 1990 |
| | | ↑ | Katz *et al.*, 1990*a* |
| | | ↑ | Chao *et al.*, 1990 |
| | | ↑ | Chao *et al.*, 1991 |
| | | ↑ | Harrison *et al.*, 1990 |
| Ara-C[a] | DNA repair/ polymerase α | ↑ | Trujillo & Yang, 1989 |
| | | ↑/– | Trujillo *et al.*, 1989 |
| | | ↑ | Berek *et al.*, 1989 |
| | | – | Howell & Gill, 1985 |
| | | – | Kyriazis *et al.*, 1985 |
| Ara-C[a] + caffeine | DNA repair/ polymerase α | ↑ | Kyriazis *et al.*, 1985 |
| Ara-C[a] + hydroxyurea | DNA repair/ polymerase α | ↑ | Swinnen *et al.*, 1989 |
| AZT[b] | Thymidine kinase/ polymerases | ↑ | Nyce *et al.*, 1990 |
| | | ↑ | Scanlon *et al.*, 1989*a* |
| 5-FU[c] | TTP[d] synthesis | ↑ | Scanlon *et al.*, 1986 |
| | | ↑ | Johnston & Allegra, 1990 |

[a]1-β-D-arabinofuranosylcytosine. [b]Azidothymidine. [c]5-Fluorouracil. [d]Thymidinetriphosphate.

against other tumour types, although the magnitude of response varied among different colon lines. This specificity might be an indication of the importance of polymerase α in colon tumours that are intrinsically insensitive to various chemotherapeutic agents. Reports of a clinical trial of Ara-C and CDDP in patients with colon cancer showed a promising response rate, with acceptable toxicity (Pasccon *et al.*, 1990). The combination of Ara-C plus hydroxyurea followed by CDDP was also tested as a phase I regimen. Nephrotoxicity was dose limiting, but responses were seen in patients pretreated with CDDP, at achievable doses (Albain *et al.*, 1990).

Also for other nucleoside analogues CDDP enhancing capacities were described, probably caused by their influence on DNA repair. Recently the potentiation of CDDP by azidothymidine (AZT), a thymidine analogue was described in a human colonic adenocarcinoma cell line (Nyce *et al.*, 1990; Scanlon *et al.*, 1989*b*) and in A2780 and A2780DDP (Scanlon *et al.*, 1990). Effects were more pronounced in the resistant lines due to increased activity of thymidine kinase and polymerase β in these cell lines. Increased thymidine kinase activity, means more AZT phosphorylated to AZTTP, this increased amount of AZTTP leads to more efficient inhibition of polymerase β.

The combination of CDDP followed by 5-FU *in vitro* showed synergistic toxicity in a human ovarian carcinoma cell line (Scanlon *et al.*, 1986). This was explained by the increased amount of cellular folates found after CDDP incubation, that might enhance the inhibition of thymidilate synthase by 5-FU (Scanlon *et al.*, 1986). In a human colon carcinoma cell line also synergism of the combination CDDP/5-FU, but in reversed sequence, was observed (Johnston & Allegra, 1990), with no difference in thymidilate synthase activity nor in the binding capacity of the enzyme. But DNA damage caused by CDDP/5-FU was increased compared to CDDP alone (Johnston & Allegra, 1990).

BSO is also capable to inhibit DNA repair, because of its effect on the cellular GSH pool. In a study by Lai *et al.*, BSO induced GSH reduction inhibited DNA repair, possibly by destabilising the DNA repair enzymes or by reduction of the deoxyribonucleotide triphosphate pools (Lai *et al.*, 1989). In both GLC4 and GLC4-CDDP BSO preincubation was capable of annihilation of total Pt-DNA repair (Meijer *et al.*, 1990*b*).

### *Agents with an effect on poly(adenosine diphosphate ribosyl)ation (Table VII)*

Important for the DNA repair processes is the enzyme poly(adenosine diphosphate ribose) polymerase. Chen *et al.* demonstrated that the inhibition of poly(ADP-ribosyl)ation with nicotinamide or 3-aminobenzamide increased CDDP cytotoxicity in Ehrlich ascites carcinoma and sarcoma 180 cells implanted in mice (Chen & Pan, 1988). Also the reversal of CDDP resistance in a rat ovarian carcinoma cell line *in vitro* (Chen & Zeller, 1990*b*; Zeller *et al.*, 1991), and of the same tumour implanted in nude mice was found (Chen & Zeller, 1990*a*). In GLC4 and GLC4-CDDP coincubation for 4 days with 2, 4 or 5 mM 3-aminobenzamide did not enhance CDDP cytotoxicity, nor did preincubation with 0.5 $\mu g\ ml^{-1}$ 6-nicotinamide followed by incubation with 2 mM-3-aminobenzamide plus CDDP.

Metoclopramide, a N-substituted carboxamide derivative of benzamide, is used in the clinic as antiemetic drug. It stimulated ADP-ribosylation in normal mononuclear leukocytes *in vitro* (Pero *et al.*, 1989), and sensitised a human squamous cell carcinoma of the head and neck xenografted in mice for CDDP (Kjellén *et al.*, 1989). Metoclopramide at a clinical achievable dose of 2 $mg\ kg^{-1}$ plus CDDP reduced the amount of metastases of a murine lung adenocarcinoma xenografted in mice more than CDDP alone (Tyson *et al.*, 1990).

### *Topoisomerase II affecting agents (Table VII)*

Elevated activity of topoisomerase II was found in a nitrogen mustard resistant Burkitt's lymphoma cell line (Tan *et al.*, 1987). This enzyme is involved in DNA conformation and its activity affects replication, translation and possibly repair. Efforts were made to evaluate the importance of this enzyme in CDDP resistance and to enhance alkylating agent and CDDP cytotoxicity with topoisomerase II inhibitors, such as novobiocin and nalidixic acid. In GLC4-CDDP (De Jong *et al.*, 1990) and a CDDP resistant subline of murine leukaemia (Waud *et al.*, 1991), topoisomerase II activity was increased compared to their sensitive mother lines. Novobiocin increased CDDP cytotoxicity *in vitro* in Chinese hamster ovary (CHO) cells, in a epipodophyllotoxin (VP16) resistant CHO subline (Eder *et al.*, 1990) and *in vivo* in FSaIIC fibrosarcoma

**Table VII** Agents affecting other nuclear and DNA related systems

| *Modulator* | *Mechanism affected* | *Cytotoxicity* | *References* |
|---|---|---|---|
| 3-Amino-benzamide | Poly)ADP-ribosyl)ation | ↑ | Chen & Pan, 1988 |
| | | ↑ | Zeller *et al.*, 1991 |
| | | ↑ | Chen & Zeller, 1990*a* |
| | | ↑ | Chen & Zeller, 1990*b* |
| Metoclo-pramide | Poly)ADP-ribosyl)ation | ↑ | Kjellén *et al.*, 1989 |
| | | ↑ | Tyson *et al.*, 1990 |
| Novobiocin | Topoisomerase II | ↑ | Eder *et al.*, 1987 |
| | | ↑ | Eder *et al.*, 1989 |
| | | ↑ | Eder *et al.*, 1990 |
| | | ↑/– | De Jong *et al.*, 1990 |
| | | ↑ | De Jong *et al.*, 1991 |
| | | ↑ | Sriram *et al.*, 1990 |
| | | – | Katz *et al.*, 1990*b* |
| DFMO[a] | DNA accessibility | ↑ | Allen & Natal, 1986 |
| | | ↑ | Chang *et al.*, 1987 |
| | | ↓ | Oredsson *et al.*, 1982 |
| | | ↑ | Hunter *et al.*, 1990 |
| Hyperthermia | DNA accessibility | ↑ | Meyn *et al.*, 1980 |
| Docosahexaenoic acid | DNA accessibility | ↑/– | Timmer-Bosscha *et al.*, 1989 |

[a]α-Difluoromethylornithine.

(Eder *et al.*, 1989; Eder *et al.*, 1987). This potentiation was also observed in GLC4 and GLC4-CDDP after short, high novobiocin incubations (De Jong *et al.*, 1991) but novobiocin had this effect only in GLC4 after continuous incubations with low concentrations (De Jong *et al.*, 1990). In a human mesothelioma and a breast cancer cell line (Sriram *et al.*, 1990) as well as in GLC4 and GLC4-CDDP (De Jong *et al.*, 1991) novobiocin incubation led to an increase in DNA interstrand cross-links. In a CDDP resistant human ovarian carcinoma cell line there was no effect of novobiocin modulation (Katz *et al.*, 1990*b*). Incubation with $0.5\,\mu$-g ml$^{-1}$ nalidixic acid, started 3 h before CDDP addition and continued during a 4 days culture did not increase CDDP sensitivity of GLC4 and GLC4-CDDP. In a phase I trial of novobiocin and cyclophosphamide, serum levels could be acheived that *in vitro* and *in vivo* in mice were sufficient to achieve cyclophosphamide potentiation. Partial response and stable disease were observed in patients who had progression on prior cyclophosphamide combination therapy (Eder *et al.*, 1991). These results may encourage the start of a trial of novobiocin and CDDP.

Another example of affecting CDDP toxicity via topoisomerase II modulation could be the improved *in vivo* activity that was observed when CDDP was combined with VP16 (Schlabel *et al.*, 1979; Sculier & Klastersky, 1984; Bosl *et al.*, 1985) an anticancer drug that forms so called cleavable complexes with topoisomerase II. Cellular, mechanistic, synergism of CDDP and VP16 could not be identified by Tsai *et al.* after extensive *in vitro* experiments and statistical calculations (Tsai *et al.*, 1989). They suggested that the apparent synergistic improvement of the *in vivo* therapeutic index of CDDP combined with VP16 was due to nonoverlapping toxicities. But an alternating regimen of first CDDP followed by VP16 showed increased activity in V79 multicell spheroids: cells were recruited into active proliferation by CDDP, after which VP16 treatment was much more effective (Durand & Vanderbyl, 1990). This recruitment could play a role in tumours with a high growth fraction.

The determination of higher levels of DNA repair enzymes started only recently. Of earlier developed CDDP resistant cell lines no information about repair enzyme levels is available. The importance of these increased activities is therefore not yet clear. However when the role of the DNA repair enzymes can be confirmed by future investigations, their detection and quantitation with the use of specific antibodies and probes against their mRNAs might be a way to determine tumour cell resistance in tumour biopsies. In addition this repair would offer a promising target for modulation.

### *DNA accessibility modulators (Table VII)*

In addition to direct inhibition of enzymes involved with repair, effects on DNA accessibility and on repair systems can be achieved via interference with the DNA conformational state. Changes in conformation are often suggested as a possible mechanism of resistance, until now little is known about the relevance of this phenomenon.

Polyamines are involved in conformation of DNA. Their equilibrium can be modulated with α-difluoromethylornithine, a specific inhibitor of ornithine decarboxylase. Coincubation with difluoromethylornithine resulted in augmentation (Allen & Natal, 1986; Chang *et al.*, 1987) as well as reduction (Oredsson *et al.*, 1982; Hunter *et al.*, 1990) of CDDP cytotoxicity. But results are variably and highly schedule dependent.

Hyperthermia appears to have, apart from effect on CDDP accumulation, an effect on Pt-DNA cross-link formation (Meyn *et al.*, 1980; Herman *et al.*, 1988). Meyn *et al.* reported increased cross-link formation in CHO cells, after treatment with CDDP at 43°C. This might have been due to increased accumulation, although this was not measured. *In vitro*, temperatures of 42°C and higher increased the rate of the reaction of CDDP with pBR322 plasmid DNA (Herman *et al.*, 1988). There are some data available that show that CDDP resistant cells are not cross resistant with heat (Wallner *et al.*, 1986; Mansouri *et al.*, 1989; GLC4-CDDP unpublished data) therefore the combination of hyperthermia and CDDP can be of clinical significance. When heat was used as total body treatment in rats CDDP induced nephrotoxicity increased, although with optimal heat/drug scheduling an improved therapeutic index was obtained (Baba *et al.*, 1989). In mice bearing a murine mammary carcinoma the more severe side effects of CDDP combined with local hyperthermia could be reduced by diethyldithiocarbamate (Murthy *et al.*, 1987), an agent known to diminish CDDP host toxicity (Rao *et al.*, 1985). Clinical studies with the combination of local, intraluminal hyperthermia combined with CDDP (Li & Hou, 1987) or with CDDP plus radiation (Hou *et al.*, 1989) for the treatment of oesophageal cancer showed promising results.

An influence of the polyunsaturated fatty acid docosahexaenoic acid (DCHA) on nuclear factors such as DNA conformation was suggested in GLC4-CDDP: after exposure to this agent increased numbers of interstrand cross-links were produced by CDDP, in correlation with cytotoxicity in this resistant line. This effect was not observed in its sensitive mother line GLC4, although accumulation increased in both lines (Timmer-Bosscha *et al.*, 1989). For DCHA it was found that oral administration could bring about changes in human leukocyte fatty acid composition (Lee *et al.*, 1985), and in rats changes of tumour cell fatty acid composition were found (Karmali *et al.*, 1984).

Although DNA conformation can play a role in resistance, changes in DNA conformation cannot be detected easily, making them an unlikely parameter, in a search for clinically feasible detection and modulation of CDDP resistance. However, modulators affecting for instance topoisomerase II may, via this enzyme, also indirectly be DNA conformation modulators.

### CDDP analogues

The most widely studied CDDP analogues until now are carboplatin (CBCDA, cis-diamminecyclobutane-1,1-dicarboxylatoplatinum(II)) and iproplatin (CHIP, cis-dichloro-bis-isopropylaminetranshydroxy-platinum(IV)) (for review: Foster *et al.*, 1990). These compounds showed an antitumour activity similar to CDDP, but were less potent than CDDP (Foster *et al.*, 1990). In CDDP resistant cells, *in vitro*, there was in general partial or complete cross resistance for both derivatives (for review: De Graeff *et al.*, 1988). The rationale for the ongoing clinical development of carboplatin and iproplatin is that both compounds showed less renal and gastrointestinal toxicity than CDDP. For both drugs bone marrow toxicity was dose limiting (Foster *et al.*, 1990). In new series of analogues the identification of agents that show activity in tumours resistant to CDDP should have priority.

The 1,2-diamminecyclohexaneplatinum (DACH-Pt) derivatives showed little or no cross resistance in a variety of CDDP resistant cell lines (for review: De Graeff *et al.*, 1988). Studies with CDDP resistant and sensitive murine leukaemia (Kraker & Moore, 1988*a*), human ovarian and human colon carcinoma cell lines (Schmidt & Chaney, 1991) showed that cells with decreased CDDP accumulation were not defective in DACH-Pt accumulation. In the same cell lines it was demonstrated that DACH-Pt-DNA adducts were less well tolerated than CDDP (Schmidt & Chaney, 1991) and ethylene diammine-Pt formed DNA adducts (Gibbons *et al.*, 1991). The latter compound is supposed to behave cellularly comparable with CDDP (Eastman, 1983). Repair of DACH-Pt induced DNA damage was increased in the CDDP resistant murine cell line (Gibbons *et al.*, 1991), but not in resistant human lines (Schmidt & Chaney, 1991). In both the CDDP resistant murine and human lines repair of CDDP induced DNA lesions was increased (Schmidt & Chaney, 1991; Gibbons *et al.*, 1991). This indicates a role for differences in accumulation, adduct toxicity and repair in the non-cross resistance of DACH-Pt analogues. As accumulation defects and increased DNA repair are prevalent mechanisms of CDDP resistance, DACH-Pt derivatives could be a worthwhile alternative in the treatment of CDDP refractory tumours. Clinical studies on their applicability have recently been started.

Third generation platinum analogues that might be an alternative for the treatment of CDDP refractory tumours are for instance lobaplatin (D19466, 1,2-bisamminomethyl-cyclobutaneplatinum(II)-lactate) and enloplatin (CL 287,110; (SP-4-2)-[1,1-cyclobutanedicarboxylato (2)-O,O'] (tetrahydro-4H-pyran-4,4-dimethanamine-N,N') platinum). For lobaplatin only partial cross resistance was found in a CDDP resistant murine leukaemia cell line (Voegeli *et al.*, 1990). In our laboratory we observed cross resistance for lobaplatin in GLC4-CDDP, but none in a CDDP resistant teratocarcinoma cell line (Meijer *et al.*, 1991). This drug showed activity in CDDP resistant ovarian cancer in a recent phase I study (Gietema *et al.*, 1991). Enloplatin was active against CDDP resistant xenografts of murine leukaemia, and more active than CDDP against a colon adenocarcinoma in mice (Durr *et al.*, 1991). Only partial cross resistance was observed in GLC4-CDDP and in a CDDP resistant teratocarcinoma cell line (Meijer *et al.*, 1991). In the clinic for lobaplatin no nephrotoxicity was observed (Gietema *et al.*, 1991).

### Conclusions

CDDP resistance as found in the clinic will be multifactorial. Interactions between several mechanisms identified *in vitro* such as signal transduction alterations, oncogenic expression and activities of DNA repair enzymes have been suggested (Andrews & Howell, 1990; Scanlon *et al.*, 1989*a*). Further research to establish the identity of such resistance cascades *in vitro* should be an objective for future research. Finding the interconnection between resistance mechanisms might facilitate cancer treatment in more than one way. A cascade will indicate a starting point in the development of resistance, an early event that might serve as a focus for the detection of tumour unresponsiveness. In contrast patient material will probably show all stages of resistance development in one tumour.

Analysis *in vitro* will also indicate the most relevant modulator(s) that can circumvent tumour resistance, as interference will be most adequate if it attacks the rate limiting step in a chain. According to Scanlon (Scanlon *et al.*, 1989*a*) the inhibition of the TTP synthesis cycle, the rate limiting step in the generation of deoxynucleotides for DNA synthesis, might substantially affect the increased DNA repair in various CDDP resistant cells. On the other hand combination of modulators, required for multifactorial CDDP resistance, can be carried out more efficiently once knowledge of the relevant interactions is gained. Apart from this, it should be considered for all combined therapies, whether *in vitro* or *in vivo* that potentiation needs thorough statistical analysis to distinguish between additivity and supra-additivity or synergism (Steel & Peckham, 1979). For VP16 for instance statistical analysis revealed no biochemical synergism with CDDP (Tsai *et al.*, 1990). In a lot of other studies due to the limited number of concentrations of the modulator used, these extensive calculations could not be made. Based on the article of Steel and Peckham (1979), conclusions in these studies should be restricted to increased or not increased, without further specification.

For clinical use optimal doses of these modulators will have to be established. For the CDDP cytotoxicity increasing anticancer drugs described in this review, Ara-C, 5-FU, and VP16 the maximal tolerable doses in combination with CDDP are well defined. For most of the other drugs required doses still need to be established and until now published trials with CDDP modulators are equivocal as far as therapeutic results are concerned.

However unexpected mechanisms may play a role *in vivo*: Teicher *et al.* developed CDDP resistance *in vivo* in mice with EMT6 murine mammary tumours. Although *in vivo* a highly resistant tumour was obtained, no *in vitro* CDDP resistance of the cell lines derived from this tumour was observed. The elimination of CDDP in the resistant EMT6 bearing mice differed from that in the mice bearing the sensitive cells. As a consequence the area under the curve of CDDP serum concentration *vs* time of mice bearing EMT6/CDDP was dramatically decreased, indicating the production of cofactors that enhance clearance of the drug by the resistant cells (Teicher *et al.*, 1990). Such factors could complicate attempts of modulation of resistance in the clinic. The mechanisms underlying this type of resistance and its significance remain however to be established. Especially in this type of resistance the use of CDDP analogues, that are not only non-cross resistant *in vitro*, but in addition have a different mode of clearance could be worthwhile.

In summary, for modulation of CDDP resistance in the clinic a growing number of potentially useful agents emerges from the laboratory bench. It will be important to elucidate not only resistance mechanisms, but also the possible interaction between these mechanisms. This will facilitate the detection and modulation of resistance of tumours, as the number of parameters, that need to be studied, will be reduced and the effects of (combinations of modulators) will become more predictable.

The impact of the known, potentially useful modulators on response and survival is eagerly awaited, as is the effect of third generation Platinum analogues.

### References

ALBAIN, K.S., SWINNEN, L.J., ERICKSON, L.C., STIFF, P.J. & FISHER, R.I. (1990). Cisplatin preceded by concurrent cytarabine and hydroxyurea: a pilot based on an *in vitro* model. *Cancer Chemother. Pharmacol.*, **27**, 33-40.

ALLEN, E.D. & NATAL, R.B. (1986). Effect of α-difluoromethylornithine alone and in combination with doxorubicin hydrochloride, cis-diamminedichloroplatinum (II), and vinblastine sulfate on the growth of P3J cells *in vitro*. *Cancer Res.*, **46**, 3550-3555.

ANDREWS, P.A & ALBRIGHT, K.D. (1991). Role of membrane ion transport in cisplatin accumulation. *Proc. Sixth International Symposium on Platinum and other Metal Compounds*, San Diego: 37.

ANDREWS, P.A. & HOWELL, S.B. (1990). Cellular pharmacology of cisplatin: perspectives on mechanisms of acquired resistance. *Cancer Cells*, **2**, 35-43.

ANDREWS, P.A., MANN, S.C., HUYNH, H.H. & ALBRIGHT, K.D. (1991). Role of the $Na^+$,$K^+$-adenosine triphophatase in the accumulation of cis-diamminedichloroplatinum(II) in human ovarian carcinoma cells. *Cancer Res.*, **51**, 3677-3681.

ANDREWS, P.A., MURPHY, M.P. & HOWELL, S.B. (1986). Differential sensitization of human ovarian carcinoma and mouse L1210 cells to cisplatin and melphalan by glutathione depletion. *Mol. Pharmacol.*, **30**, 643-650.

BABA, H., SIDDIK, Z.H., STREBEL, F.R., JENKINS, G.N. & BULL, J.M. (1989). Increased therapeutic gain of combined cisdiamminedichloroplatinum(II) and whole body hyperthermia therapy by optimal heat/drug scheduling. *Cancer Res.*, **49**, 7041-7044.

BARRY, M.A., BEHNKE, C.A. & EASTMAN, A. (1990). Activation of programmed cell death (apoptosis) by cisplatin, other anticancer drugs, toxins and hyperthermia. *Biochem. Pharmacol.*, **40**, 2353-2362.

BASU, A. & LAZO, J.S. (1991). Suppression of dexamethasone-induced methallothionein expression and cis-diamminedichloroplatinum (II) resistance by v-*mos*. *Cancer Res.*, **51**, 893-896.

BASU, A., TEICHER, B.A. & LAZO, J.S. (1990). Involvement of protein kinase C in phorbol ester-induced sensitization of HeLa cells to cis-diamminedichloroplatinum(II). *J. Biol. Chem.*, **265**, 8451-8457

BATIST, G., BEHRENS, B.C., MAKUCH, R., HAMILTON, T.C., KATKI, A.G., LOUIE, K.G., MEYERS, C.E. & OZOLS, R.F. (1986). Serial determinations of glutathione levels and glutathione-related enzyme activities in human tumor cells *in vitro*. *Biochem. Pharmacol.*, **35**, 2257–2259.

BERD, D., MCLAUGHLIN, C.J., HART, E., WIEBE, V.J., MASTRANGELO, M.J., BELLET, R.E. & DEGREGORIO, M.W. (1991). Short course, high-dose tamoxifen with cytotoxic chemotherapy for metastatic melanoma. *Proc. Am. Soc. Clin. Oncol.*, **10**, 291.

BEREK, J.S., SCHINK, J.C. & KNOX, R.M. (1989). Synergistic effect of combined intraperitoneal cisplatin and cytosine arabinoside in a murine ovarian cancer model. *Obstet. Gynecol.*, **741**, 663–666.

BOSL, G.J., YAGODA, A., GOLBEY, R.B., WHITMORE, W., HERR, H., SOGANI, P., MORSE, M., VOGELZAND, N. & MCDONALD, G. (1985). Role of etoposide-based chemotherapy in the treatment of patients with refractory or relapsing germ cell tumours. *Am. J. Med.*, **78**, 423–428.

CHAN, T.C.K., COPPOC, G.L., ZIMM, S., CLEARY, C. & HOWELL, S.B. (1988). Pharmacokinetics of intraperitoneally administered dipyridamole in cancer patients. *Cancer Res.*, **48**, 215–218.

CHANG, B.K., GUTMAN, R. & CHOU, T.-C. (1987). Schedule-dependent interaction of α-difluoromethylornithine and cis-diamminedichloroplatinum (II) against human and hamster pancreatic cancer cell lines. *Cancer Res.*, **47**, 2247–2250.

CHAO, C.C.-K., LEE, Y.-L. & LIN-CHAO, S. (1991). Enhanced host cell reactivation of damaged plasmid DNA in HeLa cells resistant to cis-diamminedichloroplatinum(II). *Cancer Res.*, **51**, 601–605.

CHAO, C.C.-K., LEE, Y.-L. & LIN-CHAO, S. (1990). Phenotypic reversion of cisplatin resistance in human cells accompanies reduced host cell reactivation of damaged plasmid. *Biochem. Biophys. Res. Comm.*, **170**, 851–859.

CHEN, G. & PAN, Q. (1988). Potentiation of the antitumour activity of cisplatin in mice by 3-aminobenzamide and nicotinamide. *Cancer Chemother. Pharmacol.*, **22**, 303–307.

CHEN, G. & ZELLER, W.J. (1990*a*). Enhancement of cisplatin (DDP) antitumor activity by 3-aminobenzamide in rat ovarian tumors sensitive and resistant to DDP *in vivo*. *Cancer Chemother. Pharmacol.*, **26**, 37–41.

CHEN, G. & ZELLER, W.J. (1990*b*). *In vitro* investigations on induction and reversal of cisplatin resistance in a rat ovarian tumor cell line. *J. Cancer Res. Clin. Oncol.*, **116**, 443–447.

CHRISTEN, R.D., HOM, D.K., PORTER, D.C., ANDREWS, P.A., MACLEOD, C.L., HAFSTROM, L. & HOWELL, S.B. (1990). Epidermal growth factor regulates the *in vitro* sensitivity of human ovarian carcinoma cells to cisplatin. *J. Clin. Invest.*, **86**, 1632–1640.

CHU, G. & CHANG, E. (1990). Cisplatin-resistant cells express increased levels of a factor that recognizes damaged DNA. *Proc. Natl Acad. Sci. USA*, **87**, 3324–3327.

CORNWELL, M.M., PASTAN, I. & GOTTESMAN, M.M. (1987). Certain calcium channel blockers bind specifically to multidrug-resistant human KB carcinoma membrane vesicles and inhibit drug binding to P-glycoprotein. *J. Biol. Chem.*, **262**, 2166–2170.

DE GRAEFF, A., SLEBOS, R.J.C. & RODENHUIS, S. (1988). Resistance to cisplatin and analogues: mechanisms and potential clinical implications. *Cancer Chemother. Pharmacol.*, **22**, 325–332.

DE JONG, S., TIMMER-BOSSCHA, H., DE VRIES, E.G.E. & MULDER, N.H. (1990). Increased topoisomerase II activity in a cisplatin resistant cell line. *Proc. Am. Assoc. Cancer Res.*, **31**, 337.

DE JONG, S., TIMMER-BOSSCHA, H., DE VRIES, E.G.E. & MULDER, N.H. (1991). Topoisomerase II, nuclear matrix proteins and formation of interstrand cross-links in a CDDP resistant human small cell lung carcinoma cell line. *Proc. Am. Assoc. Cancer Res.*, **32**, 361.

DORNISH, J.M., PETTERSEN, E.O. & OFTEBRO, R. (1989). Modifying effect of cinnamaldehyde and cinnamaldehyde derivatives on cell inactivation and cellular uptake of cis-diamminedichloroplatinum(II) in human NHIK 3025 cells. *Cancer Res.*, **49**, 3917–3921.

DURAND, R.E. & VANDERBYL, S.L. (1990). Schedule dependency for cisplatin and etoposide multifraction treatments of spheroids. *J. Natl Cancer Inst.*, **82**, 1841–1845.

DURR, F.E., CARVAJAL, S.G. & WALLACE, R.E. (1991). Enloplatin (CL287,110), a new platinum derivative with significant antitumor activity in mice. *Proc. Sixth International Symposium on Platinum and other Metal Compounds*, San Diego: 110.

EASTMAN, A. (1990). Activation of programmed cell death by anticancer agents: cisplatin as a model system. *Cancer Cells*, **2**, 275–280.

EASTMAN, A. (1983). Characterization of the adducts produced in DNA by cis-diamminedichloroplatinum(II) and cis-dichloro (ethylenediammine)platinum(II). *Biochemistry*, **22**, 3927–3933.

EDER, J.P., TEICHER, B.A., HOLDEN, S.A., CATHCART, K.N.S., SCHNIPPER, L.E. & FREI III, E. (1989). Effect of novobiocin on the antitumor activity and tumor cell and bone marrow survivals of three alkylating agents. *Cancer Res.*, **49**, 595–598.

EDER, J.P., TEICHER, B.A., HOLDEN, S.A., CATHCART, K.N.S. & SCHNIPPER, L.E. (1987). Novobiocin enhances alkylating agent cytotoxicity and DNA interstrand cross-links in a murine model. *J. Clin. Invest.*, **79**, 1524–1528.

EDER, J.P., TEICHER, B.A., HOLDEN, S.A., SENATOR, L., CATHCART, K.N.S. & SCHNIPPER, L.E. (1990). Ability of four potential topoisomerase II inhibitors to enhance the cytotoxicity of cis-diamminedichloroplatinum II in chinese hamster ovary cells and in an epipodophylltoxin-resistant subline. *Cancer Chemother. Pharmacol.*, **26**, 423–428.

EDER, J.P., WHEELER, C.A., TEICHER, B.A. & SCHNIPPER, L.E. (1991). A phase I clinical trial of novobiocin, a modulator of alkylating agent cytotoxicity. *Cancer Res.*, **51**, 510–513.

EICHHOLTZ-WIRTH, H. & HIETEL, B. (1990). Heat sensitization to cisplatin in two cell lines with different drug sensitivites. *Int. J. Hyperthermia*, **6**, 47–55.

FARNWORTH, P.G., HILLCOAT, B.L. & ROOS, I.A.G. (1989). Metallothionein induction in mouse tissues by cis-diamminedichloroplatinum(II) and its hydrolysis products. *Chem.-Biol. Interactions*, **69**, 319–332.

FOSTER, B.J., HARDING, B.J., WOLPERT-DEFILIPPES, M.K., RUBINSTEIN, L.Y., CLAGETT-CARR, K. & LEYLAND-JONES, B. (1990). A strategy for the development of two clinically active cisplatin analogs: CBDCA and CHIP. *Cancer Chemother. Pharmacol.*, **25**, 395–404.

FRAM, R.J., ROBICHAUD, N., BISHOV, S.D. & WILSON, J.M. (1987). Interactions of cis-diamminedichloroplatinum (II) with 1-β-D-arabinofuranosylcytosine in LoVo colon carcinoma cells. *Cancer Res.*, **47**, 3360–3365.

GIBBONS, G.R., PAGE, J.D., MAULDIN, S.K., HUSAIN, I. & CHANEY, S.G. (1991). Role of carrier ligand in platinum resistance in L1210 cells. *Cancer Res.*, **50**, 6497–6501.

GIETEMA, J.A., AULENBACHER, P., DE VRIES, E.G.E., UGES, D.R.A., GUCHELAAR, H.J., WILLEMSE, P.H.B., SLEIJFER, D.Th. & MULDER, N.H. (1991). A phase I study of 1,2-diamminomethylcyclobutanplatinum(II)-lactate (D19466). *Proc. Am. Soc. Clin. Oncol.*, **10**, 100.

HAMILTON, T.C., WINKER, M.A., KOUIE, K.G., BATIST, G., BEHRENS, B.C., TSURUO, T., GROTZINGER, K.R., MCKOY, W.M., YOUNG, R.C. & OZOLS, R.F. (1985). Augmentation of adriamycin, melphalan and cisplatin cytotoxicity in drug-resistant and -sensitive human ovarian carcinoma cell lines by buthionine sulfoximine mediated glutathione depletion. *Biochem. Pharmacol.*, **34**, 2583–2586.

HARRISON, S.D. Jr, HAMILTON, T.C., DYKES, D.J., WAUD, W.R. & GRISWOLD, D.P. Jr (1990). Modulation of cisplatin cytotoxicity by aphidicolin glycinate in human ovarian cancer xenografts. *Proc. Am. Assoc. Cancer Res.*, **31**, 446.

HERMAN, T.S., TEICHER, B.A., CHAN, V., COLLINS, L.S., KAUFMANN, M.E. & LOH, C. (1988). Effedt of hyperthermia on the action of cis-diamminedichloroplatinum(II), rhodamine $123_2$[tetrachloroplatinum(II)], rhodamine 123, and potassium tetrachloroplatinate *in vitro* and *in vivo*. *Cancer Res.*, **48**, 2335–2341.

HOFMANN, J., DOPPLER, W., JAKOB, A., MALY, K., POSCH, L., UBERALL, F. & GRUNICKE, H.H. (1988). Enhancement of the antiproliferative effect of cis-diamminedichloroplatinum(II) and nitrogen mustard by inhibitors of protein kinase C. *Int. J. Cancer*, **42**, 382–388.

HOFMANN, J., FIEBIG, H.H., WINTERHALTER, B.R., BERGER, D.P. & GRUNICKE, H. (1990). Enhancement of the antiproliferative activity of cis-diamminedichloroplatinum (II) by quercetin. *Int. J. Cancer*, **45**, 536–539.

HOFMANN, J., UEBERALL, F., POSCH, L., MALY, K., HERMANN, D.B.J. & GRUNICKE, H. (1989). Synergistic enhancement of the antiproliferative activity of cis-diamminedichloroplatinum(II) by the ether lipid analogue BM 41440, an inhibitor of protein kinase C. *Lipids*, **24**, 312–317.

HONG, W.-S., SAIJO, N., SASAKI, Y., MINATO, K., NAKANO, H., NAKAGAWA, K., FUJIWARA, Y., NOMURA, K. & TWENTYMAN, P.R. (1988). Establishment and characterization of cisplatin-resistant sublines of human lung cancer cell lines. *Int. J. Cancer*, **42**, 462–467.

HOSPERS, G.A.P., MULDER, N.H., DE JONG, B., DE LEIJ, L., UGES, D.R.A., FICHTINGER-SCHEPMAN, A.M.J., SCHEPER, R.J. & DE VRIES, E.G.E. (1988*a*). Characterization of a human small cell lung carcinoma cell line with acquired resistance to cis-diamminedichloroplatinum(II) *in vitro*. *Cancer Res.*, **48**, 6803–6807.

HOSPERS, G.A.P., MULDER, N.H. & DE VRIES, E.G.E. (1988*b*). Mechanisms of cellular resistance to cisplatin (review). *Med. Oncol. Tumor Pharmacother.*, **5**, 145-151.

HOU, B.S., XIONG, Q.B. & LI, D.J. (1989). Thermo-chemo-radiotherapy of esophageal cancer. *Cancer*, **64**, 1777-1782.

HOWELL, S.B. & GILL, S. (1985). Lack of energy between cisplatinum and cytarabine against ovarian carcinoma. *in vitro*. *Cancer Treat. Rep.*, **70**, 409-410.

HOWELL, S.B., VICK, J., ANDREWS, P.A., VELURY, S. & SANGA, S. (1987). Dipyridamole: biochemical modulation of cisplatin. In *Proceedings of the fifth international symposium on platinum and other metal coordination compounds in cancer chemotherapy*. Marino, N. (ed.), Martinus Nijhoff Publishing: Padua, Italy, 228-234.

HROMAS, R.A., ANDREWS, P.A., MURPHY, M.P. & BURNS, C.P. (1987). Glutathione depletion reverses cisplatin resistance in murine L1210 leukemia cells. *Cancer Lett.*, **34**, 9-13.

HUNTER, K.J., DEEN, D.F., PELLARIN, M. & MARTON, L.J. (1990). Effect of α-difluoromethylornithine on 1,3-bis(2-chloroethyl)-1-nitrosourea and cis-diammine-dichloroplatinum (II) cytotoxicity, DNA interstrand cross-linking and growth in human brain tumor cell lines *in vitro*. *Cancer Res.*, **50**, 2769-2772.

IKEDA, H., NAKANO, G., NAGASHIMA, K., SAKAMOTO, K., HARASAWA, N., KITAMURA, T., NAKAMURA, T. & NAGAMACHI, Y. (1987). Verapamil enhancement of antitumor effect of cis-diamminedichloroplatinum(II) in nude mouse-grown human neuroblastoma. *Cancer Res.*, **47**, 231-234.

ISONISHI, S., ANDREWS, P.A. & HOWELL, S.B. (1990). Increased sensitivity to cis-diamminedichloroplatinum(II) in human ovarian carcinoma cells in response to treatment with 12-*O*-tetradecanoylphorbol-13-acetate. *J. Biol. Chem.*, **265**, 3623-3627.

ISSANDOU, M., FAUCHER, C., BAYARD, F. & DARBON, J.M. (1990). Opposite effects of tamoxifen on *in vitro* protein kinase C activity and endogenous protein phosphorylation in intact MCF-7 cells. *Cancer Res.*, **50**, 5845-5850.

JOHNSTON, P. & ALLEGRA, C. (1990). The interaction of 5-FU and cisplatin in human colon carcinoma cells. *Proc. Am. Assoc. Cancer Res.*, **31**, 421.

KARMALI, R.A., MARSH, J. & FUCHS, C. (1984). Effect of omega-3 fatty acids on growth of a rat mammary tumor. *J. Natl Cancer Inst.*, **73**, 457-461.

KARTNER, N., RIORDAN, K.R. & LING, V. (1983). Cell surface P-glycoprotein associated with multidrug resistance in mammalian cell lines. *Science*, **221**, 1285-1288.

KASAHRA, K., FUJIWARA, Y., NISHIO, K. & 5 others (1991). Metallothionein content correlates with the sensitivity of human small cell lung cancer cell lines to cisplatin. *Cancer Res.*, **51**, 3237-3242.

KASHANI-SABET, M., WANG, W. & SCANLON, K.J. (1990). Cyclosporin A suppresses cisplatin-induced c-*fos* gene expression in ovarian carcinoma cells. *J. Biol. Chem.*, **265**, 11285-11288.

KATZ, E.J., ANDREWS, P.A. & HOWELL, S.B. (1990*a*). The effect of polymerase inhibitors on the cytotoxicity of cisplatin in human ovarian carcinoma cells. *Cancer Commun.*, **2**, 159-164.

KATZ, E.J., VICK, J.S., KLING, K.M., ANDREWS, P.A. & HOWELL, S.B. (1990*b*). Effect of topoisomerase modulators on cisplatin toxicity in human ovarian carcinoma cells. *Eur. J. Cancer*, **26**, 724-727.

KAWAI, K., KAMATANI, N., GEORGES, E. & LING, V. (1990). Identification of a membrane glycoprotein overexpressed in murine lymphoma sublines resistant to cis-diamminedichloroplatinum(II). *J. Biol. Chem.*, **265**, 13137-13142.

KEANE, T.E., ROSNER, G., DONALDSON, J.T., NORWOOD, D.L., POULTON, S.H. & WALTHER, P.J. (1990). Dipyridamole-Cisplatin potentiation: enhanced *in vivo* cytotoxicity in xenograft models of human testicular and bladder cancers. *J. Urol.*, **144**, 1004-1009.

KELLEY, S.L. & ROZENCWEIG, M. (1989). Resistance to platinum compounds: mechanisms and beyond. *Eur. J. Cancer Clin. Oncol.*, **25**, 1135-1140.

KELLEY, S.L., BASU, A., TEICHER, B.A., HACKER, M.P., HAMER, D.H. & LAZO, J.S. (1988). Overexpression of metallothionein confers resistance to anticancer drugs. *Science*, **241**, 1813-1815.

KIKUCHI, Y., OOMORI, K., KIZAWA, I., HIRATA, J., KITA, T., MIYAUCHI, M. & KATO, K. (1987). Enhancement of antineoplastic effects of cisplatin by calmodulin antagonists in nude mice bearing human ovarian carcinoma. *Cancer Res.*, **47**, 6459-6461.

KJELLÉN, E., WENNERBERG, J. & PERO, R. (1989). Metoclopramide enhances the effect of cisplatin on xenografted squamous cell carcinoma of the head and neck. *Br. J. Cancer*, **59**, 247-250.

KRAKER, A.J. & MOORE, C.W. (1988*a*). Accumulation of cis-diamminedichloroplatinum(II) and platinum analogues by platinum-resistant murine leukemia cells *in vitro*. *Cancer Res.*, **48**, 9-13.

KRAKER, A.J. & MOORE, C.W. (1988*b*). Elevated DNA polymerase beta activity in a cis-diamminedichloroplatinum (II) resistant P388 murine leukemia cell line. *Cancer Lett.*, **38**, 307-314.

KRIS, M.G., GRALLA, R.J., TYSON, L.B., CLARK, R.A., KELSEN, D.P., REILLY, R.N., GROSHEN, S., BOSL, G.J. & KALMAN, L.A. (1985). Improved control of cisplatin-induced emesis with high-dose metoclopramide and with combinations of metoclopramide, dexamethasone, and dipenhydramine. *Cancer*, **55**, 527-534.

KUPPEN, P.J.K., SCHUITEMAKER, H., VAN'T VEER, L.J., DE BRUIJN, E.A., VAN OOSTEROM, A.T. & SCHRIER, P.I. (1988). Cis-diamminedichloroplatinum(II)-resistant sublines derived from two human ovarian tumor cell lines. *Cancer Res.*, **48**, 3355-3359.

KYRIAZIS, A.P., KYRIAZIS, A.A. & YAGODA, A. (1985). Enhanced therapeutic effect of cis-diamminedichloroplatinum (II) against nude mouse grown human pancreatic adenocarcinoma when combined with 1-β-D-arabinofuranosylcytosine and caffeine. *Cancer Res.*, **45**, 6083-6087.

LACRETA, F., BRENNAN, J., PADAVIC, K., HAMILTON, T., TEW,K., YOUNG, R., COMIS, R., OZOLS, R. & O'DWYER, P. (1991). Phase I clinical, biochemical, and pharmacokinetic study of buthionine sulfoximine (BSO) in combination with melphalan. *Proc. Am. Soc. Clin. Oncol.*, **10**, 104.

LAI, G., OZOLS,. R.F., SMYTH, J.F., YOUNG, R.C. & HAMILTON, T.C. (1988). Enhanced repair as a mechanism of resistance to cis-diamminedichloroplatinum(II). *Biochem. Pharmacol.*, **37**, 4597-4600.

LAI, G.-M., OZOLS, R.F., YOUNG, R.C. & HAMILTON, T.C. (1989). Effect of glutathione on DNA repair in cisplatin-resistant human ovarian cancer cell lines. *J. Natl Cancer Inst.*, **81**, 535-539.

LEE, T.H., HOOVER, R.L., WILLIAMS, J.D., SPERLING, R.I., RAVALESE, J., SPUR, B.W., ROBINSON, D.R., COREY, E.J., LEWIS, R.A. & AUSTEN, K.F. (1985). Effect of dietary enrichment with eicosapentaenoic and docosahexaenoic acids on *in vitro* neutrophil and monocyte leukotriene generation and neutrophil function. *N. Engl. J. Med.*, **312**, 1217-1224.

LI, D.J. & HOU, B.S. (1987). Preliminary report on the esophageal cancer by intraluminal microwave hyperthermia and chemotherapy. *Cancer Treat. Rep.*, **71**, 1013-1019.

LOEHRER, P.J. & EINHORN, L.H. (1984). Cisplatin. *Ann. Intern. Med.*, **100**, 704-713.

MANN, S.C., ANDREWS, P.A. & HOWELL, S.B. (1991). Modulation of cis-diamminedichloroplatinum(II) accumulation and sensitivity by forskolin and 3-isobutyl-1-methylxanthine in sensitive and resistant human ovarian carcinoma cells. *Int. J. Cancer*, **48**, 866-872.

MANSOURI, A., HENLE, K.J., BENSON, A.M., MOSS, A.J. & NAGLE, W.A. (1989). Characterization of a cisplatin-resistant subline of murine RIF-1 cells and reversal of drug resistance by hyperthermia. *Cancer Res.*, **49**, 2674-2678.

MASUDA, H., OZOLS, R.F., LAI, G.-M., FOJO, A., ROTHENBERG, M. & HAMILTON, T.C. (1988). Increased DNA repair as a mechanism of acquired resistance to cis-diamminedichloroplatinum(II) in human ovarian cancer cell lines. *Cancer Res.*, **48**, 5713-5716.

MASUDA, H., TANAKA, T., MATSUDA, H. & KUSABA, I. (1990). Increased removal of DNA-bound platinum in a human ovaian cancer cell line resistant to cis-diamminedichloroplatinum(II). *Cancer Res.*, **50**, 1863-1866.

MCCLAY, E.F., ALBRIGHT, K., JONES, J., CHRISTEN, R. & HOWELL, S.B. (1991). Modulation of cisplatin sensitivity by tamoxifen in human malignant melanoma. *Proc. Am. Soc. Clin. Oncol.*, **10**, 291.

MCCLAY, E.F., MASTRANGELO, M.J., SPRANDIO, J.D., BELLET, R.E. & BERD, D. (1989). The importance of tamoxifen to a cisplatin-containing regimen in the treatment of metastatic melanoma. *Cancer*, **63**, 1292-1295.

MEIJER, C., MULDER, N.H. & DE VRIES, E.G.E. (1990*a*). The role of detoxifying systems in resistance of tumor cells to cisplatin and adriamycin. *Cancer Treat. Rev.*, **17**, 389-407.

MEIJER, C., MULDER, N.H., HOSPERS, G.A.P., UGES, D.R.A. & DE VRIES, E.G.E. (1990*b*). The role of glutathione in resistance in a human small cell lung cancer cell line. *Br. J. Cancer*, **62**, 72-77.

MEIJER, C., MULDER, N.H., TIMMER-BOSSCHA, H., MEERSMA, G.J. & DE VRIES, E.G.E. (1991). Role of GSH in the efficacy of 7 Platinum compounds in 2 cisplatin resistant human cell lines. *Proc. Am. Assoc. Cancer Res.*, **32**, 408.

MEYN, R.E., CORRY, P.M., FLETCHER, S.E. & DEMETRIADES, M. (1980). Thermal enhancement of DNA damage in mammalian cells treated with cis-diamminedichloroplatinum. *Cancer Res.*, **40**, 1136-1139.

MORGAN, R.J. Jr, DOROSHOW, J.H., FLANAGAN, B., AKMAN, S., FORMAN, S., HARRISON, J., LEONG, L., MARGOLIN, K., NILAND, J., RASCHKO, J., SOMLO, G. & SCANLON, K. (1991). Phase I chemomodulation trial utilizing carboplatin with infusional cyclosporine. *Proc. Am. Soc. Clin. Oncol.*, **10**, 94.

MURTHY, M.S., RAO, L.N., KHANDEKAR, J.D. & SCANLON, E.F. (1987). Enhanced therapeutic efficacy of cisplatin by combination with diethyldithiocarbamate and hyperthermia in a mouse model. *Cancer Res.*, **47**, 774–779.

NAGOURNEY, R.A., MESSENGER, J.C., KERN, D.H. & WEISENTHAL, L.M. (1990). Enhancement of anthracycline and alkylator cytotoxicity by ethacrynic acid in primary cultures of human tissues. *Cancer Chemother. Pharmacol.*, **26**, 318–322.

NYCE, J., KLANN, R., HOLBROOK, T., MYLOTT, D. & LEONARD, S. (1990). AZT modulation of cisplatin cytotoxicity in HT 29 human colonic adenocarcinoma cells. *Proc. Am. Assoc. Cancer Res.*, **31**, 332.

ONODA, J.M., NELSON, K.K., PILARSKI, S.M., WHITE, N.S., MIHU, R.G. & HOHN, K.V. (1990). Combination chemotherapy with cisplatin and nifedipine: synergistic antitumor effects against a cisplatin-resistant subline of the B16 amelanotic melanoma. *Clin. Exp. Metastasis*, **8**, 59–73.

ONODA, J.M., NELSON, K.K., TAYLOR, J.D. & HOHN, K.V. (1989). *In vitro* characterization of combination antitumor chemotherapy with calcium channel blockers and cis-diamminedichloroplatinum (II). *Cancer Res.*, **49**, 2844–2850.

OREDSSON, S.M., DEEN, D.F. & MARTON, L.J. (1982). Decreased cytotoxicity of cis-diamminedichloroplatinum (II) by α-difluoromethylornithine depletion of polyamines in rat brain tumour cells *in vitro*. *Cancer Res.*, **42**, 1296–1299.

PASCCON, G., DIAZ, B., LITORSKA, S., NEGRO, A., MORGENFELD, E., MARANTZ, A., LEE, I., YANG, L.Y., TRUJILLO, J. & GERCOVICH, F.G. (1990). Ara C and cisplatin for advanced colon carcinoma. *Proc. Am. Assoc. Cancer Res.*, **31**, 208.

PEREZ, R.P., HANDEL, L.M., SCHILDER, R.J., OZOLS, R.F. & HAMILTON, T.C. (1990). Potentiation of cisplatin cytotoxicity by trifluoperazine, a calmodulin inhibitor. *Proc. Am. Assoc. Cancer Res.*, **31**, 403.

PERO, R.W., LYBAK, S., KJELLÉN, E. & WENNERBERG, J. (1989). Metoclopramide, a representative new class of adenosine diphosphate ribosyl transferase modulators that sensitize the cytotoxic action of drugs and radiation. *Proc. Am. Assoc. Cancer Res.*, **30**, 569.

PLUMB, J.A., MILROY, R., BICKNELL, S.R. & KAYE, S.B. (1990). Glutathione-S-transferase, P-glycoprotein and drug resistance in small cell lung cancer cell lines. *Proc. Am. Assoc. Cancer Res.*, **31**, 369.

RAO, L.N., MURTHY, M.S., KHANDEKAR, J.D. & SCANLON, E.F. (1985). Selective protection of cisplatin induced host toxicity by diethyldithiocarbamate. *Breast Cancer Res. Treat.*, **5**, 171.

RICHON, V.M., SCHULTE, N. & EASTMAN, A. (1987). Multiple mechanisms of resistance to cis-diamminedichloroplatinum(II) in murine leukemia L1210 cells. *Cancer Res.*, **47**, 2056–2061.

RINGBORG, Y., HANSSON, J., JUNGNELIUS, U., BERHANE, K., CASTRO, V. & MANNERVIK, b. (1990). Ethacrynic acid inhibtion of glutathione transferases as a mechanism for enhanced melphalan toxicity in human melanoma. *Proc. Am. Assoc. Cancer Res.*, **31**, 369.

ROBERTS, J.J. & FRIEDLOS, F. (1987). Quantitative estimation of cisplatin-induced DNA interstrand cross-links and their repair in mammalian cells: relationship to toxicity. *Pharmac. Ther.*, **34**, 215–246.

ROBICHAUD, N.J. & FRAM, R.J. (1990). Schedule dependence of buthionine sulfoximine in reversing resistance to cisplatin. *Chem.-Biol. Interactions*, **76**, 333–342.

SABURI, Y., NAKAGAWA, M., ONO, M., SAKAI, M., MARUMATSU, M., KOHNO, K. & KUWANO, M. (1989). Increased expression of glutathione S-transferase gene in cis-diamminedichloroplatinum(II)-resistant variants of a chinese hamster ovary cell line. *Cancer Res.*, **49**, 7020–7025.

SATOH, M., NAGANUMA, A. & IMURA, N. (1988). Metallothionein induction prevents toxic side effects of cisplatin and adriamycin used in combination. *Cancer Chemother. Pharmacol.*, **21**, 176–178.

SCANLON, K.J., FUNATO, T., PEZESHKI, B., TONE, T. & SOWERS, L.C. (1990). Potentiation of azidothymidine cytotoxicity in cisplatin-resistant human ovarian carcinoma cells. *Cancer Comm.*, **2**, 339–343.

SCANLON K.J., KASHANI-SABET, M., MIYACHI, H., SOWERS, L.C. & ROSSI, J. (1989*a*). Molecular basis of cisplatin resistance in human carcinomas: model systems and patients. *Anticancer Res.*, **9**, 1301–1312.

SCANLON, K.J., KASHANI-SABET, M. & SOWERS, L.C. (1989*b*). Overexpression of DNA replication and repair enzymes in cisplatin-resistant human colon carcinoma HCT8 cells and circumvention of azidothymidine. *Cancer Commun.*, **1**, 269–275.

SCANLON, K.J., NEWMAN, E.M., LU, Y. & PRIEST, D.G. (1986). Biochemical basis for cisplatin and 5-fluorouracil synergism in human ovarian carcinoma cells. *Proc. Natl Acad. Sci. USA*, **83**, 8923–8925.

SCHILDER, R.J., HALL, L., MONKS, A., HANDEL, L.M., FORNACE, A.J., OZOLS, R.F., FOJO, A.T. & HAMILTON, T.C. (1990*a*). Metallothionein gene expression and resistance to cisplatin in human ovarian cancer. *Int. J. Cancer*, **45**, 416–422.

SCHILDER, R.J., NASH, S., TEW, K., PANTING, L., COMIS, R.L. & O'DWYER, P.J. (1990*b*). Phase I trial of thiotepa in combination with the glutathione transferase inhibitor ethacrynic acid. *Proc. Am. Assoc. Cancer Res.*, **31**, 177.

SCHLABEL, F.M. Jr, TRADER, M.W., LASTER, W.R. Jr, CORBETT, T.H. & GRISWOLD, D.P. Jr (1979). Cis-dichlorodiammineplatinum (II): combination of chemotherapy and cross-resistance studies with tumors of mice. *Cancer Treat. Rep.*, **63**, 1459–1473.

SCHMIDT, W.J. & CHANEY, S.G. (1991). Characterization of carrier ligand effects on platinum resistance in two human carcinoma cell lines: cytotoxicity, uptake DNA adduct formation and repair. *Proc. Sixth International Symposium on Platinum and other Metal Compounds*, San Diego: 105.

SCULIER, J.P. & KLASTERSKY, J. (1984). Progress in chemotherapy of non-small cell lung cancer. *Eur. J. Clin. Oncol.*, **20**, 1329–1333.

SRIRAM, R., ALI-OSMAN, F., LIVINGSTON, R., ELLIS, G., NEEDLE, M.N. & STEIN, D. (1990). Modulation of topoiosomerase II on the kinetics of formation and repair of cis-platinum (cis-DDP) induced DNA-interstrand crosslinks in human tumor cells sensitive and resistant to cis-DDP. *Proc. Am. Assoc. Cancer Res.*, **31**, 335.

STEEL, G.G. & PECKHAM, M.J. (1979). Exploitable mechanisms in combined radiotherapy-chemotherapy: the concept of additivity. *Int. J. Radiation Oncol. Biol. Phys.*, **5**, 85–91.

SWINNEN, L.J., BARNES, D.M., FISHER, S.G., ALBAIN, K.S., FISHER, R.I. & ERICKSON, L.C. (1989). 1-β-D-arabinofuranosylcytosine and hydroxyurea production of cytotoxic synergy with cis-diamminedichloroplatinum (II) and modification of platinum-induced DNA interstrand cross-linking. *Cancer Res.*, **49**, 1383–1389.

TAN, K.B., MATTERN, M.R., BOYCE, R.A. & SCHEIN, P.S. (1987). Elevated DNA topoisomerase II activity in nitrogen mustard-resistant human cells. *Proc. Natl Acad. Sci. USA*, **84**, 7668–7671.

TEICHER, B.A., HERMAN, T.S., HOLDEN, S.A., WANG, Y., PFEFFER, M.R., CRAWFORD, J.W. & FREI, (III), E. (1990). Tumor resistance to alkylating agents conferred by mechanisms operative only *in vivo*. *Science*, **247**, 1457–1461.

TEICHER, B.A., HOLDEN, S.A., KELLEY, M.J., SHEA, T.C., CUCCHI, C.A., ROSOWSKY, A., HENNER, W.D. & FREI III, E. (1987). Characterization of a human squamous carcinoma cell line resistant to cis-diamminedichloroplatinum(II). *Cancer Res.*, **47**, 388–393.

TEW, K.D., BOMBER, A.M. & HOFFMAN, S.J. (1988). Ethacrynic acid and piriprost as enhancers of cytotoxicity in drug resistant and sensitive cell lines. *Cancer Res.*, **48**, 3622–3625.

TIMMER-BOSSCHA, H., HOSPERS, G.A.P., MEIJER, C., MULDER, N.H., MUSKIET, F.A.J., MARTINI, I.A., UGES, D.R.A. & DE VRIES, E.G.E. (1989). Influence of docosahexaenoic acid on cisplatin resistance in a human small cell lung carcinoma cell line. *J. Natl Cancer Inst.*, **81**, 1069–1075.

TOFFOLI, G., BEVILACQUA, C., FRANCESCHIN, A. & BOIOCCHI, M. (1989). Effect of hyperthermia on intracellular drug accumulation and chemosensitivity in drug-sensitive and drug-resistant P388 leukaemia cell lines. *Int. J. Hyperthermia*, **5**, 163–172.

TOFFOLI, G., VIEL, A., TUMIOTTO, L., BISCONTIN, G., ROSSI, C. & BOIOCCHI, M. (1991). Pleiotropic-resistant phenotype is a multifactorial phenomenon in human colon carcinoma lines. *Br. J. Cancer*, **63**, 51–56.

TRUJILLO, J.M., YANG, L.-Y., GERCOVICH, G., SU, Y.Z. & LEE, J. (1989). Metronidazole enhances the cytotoxic synergism produced by the combination of 1-β-arabino-furanosylcytosine and cis-diamminedichloroplatinum. *Anticancer Res.*, **9**, 1751–1756.

TRUJILLO, J.M. & YANG, L.-Y. (1989). Synergism of 1-β-D-arabinofuranosylcytosine and cis-diamminedichloroplatinum in their lethal efficacies against seven established cancer cell lines of gastrointestinal origin. *Anticancer Res.*, **9**, 197–202.

TSAI, C.-M., GAZDAR,M A.F., VENZON, D.J., STEINBERG, S.M., DEDRICK, R.L., MULSHINE, J.L. & KRAMER, B.S. (1989). Lack of *in vitro* synergy between etoposide and cis-diamminedichloroplatinum(II). *Cancer Res.*, **49**, 2390–2397.

TYSON, F.L., BROWN, Y.M., WALKER, D.M., BELINSKY, S.A. & ANDERSON, M.W. (1990). Inhibition of murine pulmonary metastases by cisplatinum in combination with metoclopramide. *Proc. Am. Assoc. Cancer Res.*, **31**, 388.

VASSILEV, P.M., KANAZIRSKA, M.P., CHARAMELLA, L.J., DIMITROV, N.V. & TIEN, H.T. (1987). Changes in calcium channel activity in membranes from cis-diamminedichloro-platinum (II)-resistant and -sensitive L1210 cells. *Cancer Res.*, **47**, 519–522.

VAYUVEGULA, B., SLATER, L., MEADOR, J. & GUPTA, S. (1988). Correction of altered plasma membrane potentials. A possible mechanism of cyclosporin A and verapamil reversal of pleiotropic drug resistance in neoplasia. *Cancer Chemoth. Pharmcol.*, **22**, 163–168.

VOEGELI, R., SCHUMACHER, W., ENGEL, J.P., RESPONDEK, J. & HILGARD, P. (1990). D-19466, a new cyclobutane-platinum complex with antitumor activity. *J. Cancer Res. Clin. Oncol.*, **116**, 439–442.

WALLNER, K.E., DEGREGORIO, M.W. & LI, G.C. (1986). Hyperthermic potentiation of cis-diamminedichloroplatinum(II) cytotoxicity in chinese hamster ovary cells resistant to the drug. *Cancer Res.*, **46**, 6242–6245.

WAUD, W.R., VASANTHAKUMAR, G., HARRISON, S.D. Jr, LASTER, W.R. Jr & GRISWOLD, D.P. Jr (1991). Antitumor drug cross-resistance *in vivo* in a cisplatin-resistant murine P388 leukemia. *Proc. Sixth International Symposium on Platinum and other Metal Compounds*, San Diego: 107.

ZELLER, W.J., FRÜHAUF, S., CHEN, B.K., KEPPLER, B.K., FREI, E. & KAUFMANN, M. (1991). Chemoresistance in rat ovarian tumours. *Eur. J. Cancer*, **27**, 62–67.